# Correspondence Between Perceived Pubertal Development and Hormone Levels in 9-10 Year-Olds From the Adolescent Brain Cognitive Development Study

**DOI:** 10.3389/fendo.2020.549928

**Published:** 2021-02-18

**Authors:** Megan M. Herting, Kristina A. Uban, Marybel Robledo Gonzalez, Fiona C. Baker, Eric C. Kan, Wesley K. Thompson, Douglas A. Granger, Matthew D. Albaugh, Andrey P. Anokhin, Kara S. Bagot, Marie T. Banich, Deanna M. Barch, Arielle Baskin-Sommers, Florence J. Breslin, B. J. Casey, Bader Chaarani, Linda Chang, Duncan B. Clark, Christine C. Cloak, R. Todd Constable, Linda B. Cottler, Rada K. Dagher, Mirella Dapretto, Anthony S. Dick, Nico Dosenbach, Gayathri J. Dowling, Julie A. Dumas, Sarah Edwards, Thomas Ernst, Damien A. Fair, Sarah W. Feldstein-Ewing, Edward G. Freedman, Bernard F. Fuemmeler, Hugh Garavan, Dylan G. Gee, Jay N. Giedd, Paul E. A. Glaser, Aimee Goldstone, Kevin M. Gray, Samuel W. Hawes, Andrew C. Heath, Mary M. Heitzeg, John K. Hewitt, Charles J. Heyser, Elizabeth A. Hoffman, Rebekah S. Huber, Marilyn A. Huestis, Luke W. Hyde, M. Alejandra Infante, Masha Y. Ivanova, Joanna Jacobus, Terry L. Jernigan, Nicole R. Karcher, Angela R. Laird, Kimberly H. LeBlanc, Krista Lisdahl, Monica Luciana, Beatriz Luna, Hermine H. Maes, Andrew T. Marshall, Michael J. Mason, Erin C. McGlade, Amanda S. Morris, Bonnie J. Nagel, Gretchen N. Neigh, Clare E. Palmer, Martin P. Paulus, Alexandra S. Potter, Leon I. Puttler, Nishadi Rajapakse, Kristina Rapuano, Gloria Reeves, Perry F. Renshaw, Claudiu Schirda, Kenneth J. Sher, Chandni Sheth, Paul D. Shilling, Lindsay M. Squeglia, Matthew T. Sutherland, Susan F. Tapert, Rachel L. Tomko, Deborah Yurgelun-Todd, Natasha E. Wade, Susan R. B. Weiss, Robert A. Zucker, Elizabeth R. Sowell

**Affiliations:** ^1^ Preventive Medicine, University of Southern California, Los Angeles, CA, United States; ^2^ Department of Pediatrics, Children’s Hospital Los Angeles, University of Southern California, Los Angeles, CA, United States; ^3^ Public Health, University of California, Irvine, Irvine, CA, United States; ^4^ Institute for Interdisciplinary Salivary Bioscience Research, University of California, Irvine, Irvine, CA, United States; ^5^ Department of Psychiatry, University of California, San Diego, La Jolla, CA, United States; ^6^ Research on Children, Youth, and Families, Children’s Hospital Los Angeles, University of Southern California, Los Angeles, CA, United States; ^7^ Center for Health Sciences, SRI International, Menlo Park, CA, United States; ^8^ Division of Biostatistics, University of California, San Diego, La Jolla, CA, United States; ^9^ Social Ecology, University of California, Irvine, Irvine, CA, United States; ^10^ Bloomberg School of Public Health, Johns Hopkins University, Baltimore, CA, United States; ^11^ Department of Psychiatry, University of Vermont, Burlington, VT, United States; ^12^ Department of Psychiatry, Washington University, St. Louis, MO, United States; ^13^ Department of Psychiatry, Icahn School of Medicine at Mount Sinai, New York, NY, United States; ^14^ Department of Psychology and Neuroscience, University of Colorado Boulder, Boulder, CO, United States; ^15^ Department of Psychological and Brain Sciences, Washington University, St. Louis, MO, United States; ^16^ Department of Psychology, University of Yale, New Haven, CT, United States; ^17^ Laureate Institute for Brain Research, Tulsa, OK, United States; ^18^ Diagnostic Radiology and Nuclear Medicine, University of Maryland, Baltimore, MD, United States; ^19^ Department of Psychiatry, University of Pittsburgh, Pittsburgh, PA, United States; ^20^ Radiology and Biomedical Imaging, University of Yale, New Haven, CT, United States; ^21^ Department of Epidemiology, University of Florida, Gainesville, FL, United States; ^22^ Division of Scientific Programs, National Institute on Minority Health and Health Disparities, Bethesda, MD, United States; ^23^ Psychiatry and Biobehavioral Sciences, University of California, Los Angeles, Los Angeles, CA, United States; ^24^ Department of Psychology, Florida International University, Miami, FL, United States; ^25^ Department of Neurology, Washington University, St. Louis, MO, United States; ^26^ Division of Extramural Research, National Institute on Drug Abuse, Bethesda, MD, United States; ^27^ Department of Psychiatry, University of Maryland, Baltimore, MD, United States; ^28^ Department of Pediatrics, University of Minnesota, Minneapolis, MN, United States; ^29^ Department of Psychology, University of Rhode Island, Kingston, RI, United States; ^30^ Department of Neuroscience, University of Rochester, Rochester, NY, United States; ^31^ Health Behavior and Policy, Virginia Commonwealth University, Richmon, VA, United States; ^32^ Department of Psychiatry, University of San Diego, La Jolla, CA, United States; ^33^ Psychiatry and Behavioral Sciences, Medical University of South Carolina, Charleston, SC, United States; ^34^ Department of Psychiatry, University of Michigan, Ann Arbor, MI, United States; ^35^ Center for Human Development, University of California, San Diego, La Jolla, CA, United States; ^36^ Department of Psychiatry, University of Utah, Salt Lake City, UT, United States; ^37^ Medical Cannabis & Science Program, Thomas Jefferson University, Philadelphia, PA, United States; ^38^ Department of Psychology, University of Michigan, Ann Arbor, MI, United States; ^39^ Department of Cognitive Science, University of San Diego, La Jolla, CA, United States; ^40^ Department of Physics, Florida International University, Miami, FL, United States; ^41^ Department of Psychology, University of Wisconsin, Milwaukee, WI, United States; ^42^ Department of Psychology, University of Minnesota, Minneapolis, MN, United States; ^43^ Human & Molecular Genetics, Virginia Commonwealth University, Richmond, VT, United States; ^44^ Department of Pediatrics, University of Southern California, Los Angeles, CA, United States; ^45^ Center for Behavioral Health Research, University of Tennessee, Knoxville, TN, United States; ^46^ Human Development and Family Science, Oklahoma State University, Tulsa, OK, United States; ^47^ Department of Psychiatry, Oregon Health & Science University, Portland, OR, United States; ^48^ Anatomy & Neurobiology, Virginia Commonwealth University, Richmond, VT, United States; ^49^ Department of Psychiatry, University of Utah School of Medicine, Salt Lake City, UT, United States; ^50^ Department of Radiology, University of Pittsburgh, Pittsburgh, PA, United States; ^51^ Department of Psychology, University of Missouri, Columbia, MO, United States; ^52^ Department of Psychiatry, UC San Diego, La Jolla, CA, United States

**Keywords:** adolescent brain cognitive development, salivary hormones, pubertal development scale, puberty, testosterone, dehydroepiandrosterone, estradiol

## Abstract

**Aim:**

To examine individual variability between perceived physical features and hormones of pubertal maturation in 9–10-year-old children as a function of sociodemographic characteristics.

**Methods:**

Cross-sectional metrics of puberty were utilized from the baseline assessment of the Adolescent Brain Cognitive Development (ABCD) Study—a multi-site sample of 9–10 year-olds (n = 11,875)—and included perceived physical features *via* the pubertal development scale (PDS) and child salivary hormone levels (dehydroepiandrosterone and testosterone in all, and estradiol in females). Multi-level models examined the relationships among sociodemographic measures, physical features, and hormone levels. A group factor analysis (GFA) was implemented to extract latent variables of pubertal maturation that integrated both measures of perceived physical features and hormone levels.

**Results:**

PDS summary scores indicated more males (70%) than females (31%) were prepubertal. Perceived physical features and hormone levels were significantly associated with child’s weight status and income, such that more mature scores were observed among children that were overweight/obese or from households with low-income. Results from the GFA identified two latent factors that described individual differences in pubertal maturation among both females and males, with factor 1 driven by higher hormone levels, and factor 2 driven by perceived physical maturation. The correspondence between latent factor 1 scores (hormones) and latent factor 2 scores (perceived physical maturation) revealed synchronous and asynchronous relationships between hormones and concomitant physical features in this large young adolescent sample.

**Conclusions:**

Sociodemographic measures were associated with both objective hormone and self-report physical measures of pubertal maturation in a large, diverse sample of 9–10 year-olds. The latent variables of pubertal maturation described a complex interplay between perceived physical changes and hormone levels that hallmark sexual maturation, which future studies can examine in relation to trajectories of brain maturation, risk/resilience to substance use, and other mental health outcomes.

## Introduction

Puberty is an important developmental milestone that begins with rising hormone levels and leads to physical changes in secondary sex characteristics. Puberty contributes to individual differences in brain maturation, cognition, emotion, and psychosocial wellbeing (1, 2). Individual differences in the age when physical changes emerge vary widely, with a typical onset between 8 to 13 years in females and 9 to 14 years in males (3). Moreover, variation in pubertal timing has been associated with important psychological outcomes, including risk-taking behavior (4, 5), depression symptomatology (6–9), and substance use (10–12), with more advanced physical features relating to more risk taking, depressive-like symptomatology and substance use, on average. Although there are significant implications of pubertal timing for health and wellbeing, the foundational studies that are credited for our current understanding of pubertal onset may not generalize to today’s youth, as study samples often lacked diversity and consideration of the larger sociodemographic context (3, 13–15). Thus, studies are needed to expand our basic understanding of individual differences in pubertal development in large samples of both sexes (1), as well as in narrowed age ranges, to determine the optimal approach to integrate multiple complex measures of puberty for a given individual prior to exploring relationships with developmental outcomes.

Estimates suggest that less than five percent of published studies on puberty have examined normative patterns of pubertal maturation as a function of race and ethnicity (1). While replicable differences across racial and ethnic groups have emerged, with earlier pubertal timing in participants identifying as Black or Hispanic (16–19), many studies commonly report findings in only one sex and largely fail to adequately account for sociodemographic characteristics or other key biological variables (e.g., child’s weight). However, in the United States, race and ethnicity are greatly intertwined with socioeconomic status. Further, some have postulated that racial and ethnic differences in pubertal maturation could be due to differences in weight status (18, 20, 21), given that the obesity epidemic has disproportionately impacted minority and lower-income communities (22). Being overweight or obese has been associated with earlier pubertal timing among females, but findings among males have been mixed (21, 23, 24) and are often limited by lack of sample diversity. Efforts to better understand pubertal timing must jointly consider socioeconomic status, race/ethnicity, and obesity. Only a few studies have had sufficiently large and diverse samples to begin to address combinations of these factors, but none have addressed all three factors in both sexes (18, 19, 25, 26). Therefore, the primary and overarching aim was to examine how sociodemographic characteristics relate to measures of pubertal maturation, such as perceived physical changes and hormonal features of pubertal status, in a large and diverse sample of children, all within a narrowed age range (e.g., 9.00–10.99 years old).

The transition into puberty is driven by two processes: adrenarche and gonadarche. Adrenarche is the maturation of the adrenal glands and its release of adrenal androgens, including dehydroepiandrosterone (DHEA), which contribute to the development of pubic and axillary hair as well as body odor (27). Gonadarche begins *via* release of gonadotropin releasing hormone in the hypothalamus and downstream production of gonadal steroids and the maturation of secondary sexual characteristics (28). While both testosterone and estradiol levels rise in both sexes, the magnitude of increase varies by sex, with greater increases in testosterone for males and greater increases in estradiol in females, due to additional release from the ovaries. Higher levels of testosterone promote growth of the penis and scrotum in males (29). Increases in estradiol in girls lead to breast development as well as body fat distribution, and eventually the later gonadal event of menarche (i.e., first menstrual cycle) (30). The combined rise in sex steroids, growth hormone, and insulin-like growth factor-1 (IGF-I) during puberty contribute to the adolescent growth spurt (31). Commonly used pubertal status measurements are based on visible physical features by caregiver-report, self-report, or exam by a trained clinician [see (32) for extensive review]. While hormones drive physical changes, with hormonal events preceding observable physical changes, the relationship between hormones and physical maturation may occur in a tissue-specific manner, such that different levels of hormones may be required for changes in axillary hair compared to pubic hair. Although correlated, physical maturation does not map one-to-one with hormone levels in developing individuals (32), and there is interindividual variability in timing, pubertal pathways (e.g., pubic hair vs. breast development as the initial sign), and pubertal hormone levels (33). For these reasons, there is likely not a single “gold standard” for characterizing the multifaceted process of changes in physical and hormonal markers through pubertal development (34). Thus, further studies are needed that assess and integrate individual differences in complementary physical and hormonal markers to push the field of puberty forward.

The current study leveraged multiple indicators of pubertal maturation, including perceived physical development as reported by the child and caregiver and salivary hormone levels from the child, from the large sample of 11,880 children participating in the Adolescent Brain Cognitive Development (ABCD) Study^SM^ in the U.S. (35, 36). By design, the ABCD Study^®^ enrolled 9–10 year-olds to capture the transition from childhood through adolescence (37, 38). Given the narrow age range, the ABCD Study provides an unparalleled opportunity to examine differences in pubertal status without the added complication of data manipulation and the confound of age. Moreover, the ABCD Study also provides the necessary variability and statistical power to disentangle the extent to which sex, race/ethnicity, socioeconomic, and body weight are associated with common markers of pubertal status at 9–10 years of age. Lastly, we capitalized on the potentially unique, yet complementary, information provided through integrating both perceived physical features and hormone measures by applying a group factor analysis (GFA) (39) to identify latent variables of pubertal maturation.

## Methods

### Participants and Procedure

The ABCD Study^®^ is a large-scale, 10-year longitudinal study involving 21 data collection sites across the United States (ABCDStudy.org) (35). Using school-based enrollment, community events, and birth records to identify twins, the consortium enrolled 11,880 children aged 9–10 years (38). Briefly, inclusion criteria for the ABCD study were as follows: 1) age 9.00 to 10.99 years at the time of baseline assessment; 2) able to validly and safely complete the baseline visit including MRI; 3) fluent in English. Exclusionary criteria included any of the following: a current diagnosis of schizophrenia, autism spectrum disorder (moderate, severe), mental retardation/intellectual disability, or alcohol/substance use disorder; non-correctable vision, hearing or sensorimotor impairments, as protocol elements may not be valid; major neurological disorders, such as cerebral palsy, brain tumor, stroke, brain aneurysm, brain hemorrhage, subdural hematoma, multiple sclerosis, sickle cell disease, and the following seizure disorder diagnoses: Lennox-Gastaut syndrome, Dravet syndrome, and Landau Kleffner syndrome; gestational age less than 28 weeks, and birthweight less than 1.2 kilograms (2 lb 10 oz); birth complications, other than those associated with prematurity, that resulted in being hospitalized for more than a month; a history of traumatic brain injury; or MRI contraindications. Data from 11,875 of these subjects came from the ABCD 2.0.1 data release (DOI: 10.15154/1504041), which included baseline data (i.e., cross-sectional). Centralized institutional review board (IRB) approval was obtained from the University of California, San Diego. Study sites obtained approval from their local IRBs. Written informed consent was provided by each caregiver; each child provided written assent. All ethical regulations were complied with during data collection and analysis. Child and caregiver participants’ in-person baseline visits were completed between September 2016 and October 2018.

### Self-Report Perceived Physical Maturation

In studying puberty, the measure of puberty chosen should “match” the research question and the sample under study (32, 40). Given the narrow age range, the epidemiological nature of the ABCD Study, and to minimize invasiveness, perceived physical markers of pubertal maturation were assessed by the youth and primary caregiver using the PDS scale (41). This questionnaire was also chosen as self-reports on this scale have been shown to correlate significantly with other measures of pubertal status, including physician ratings (41). The PDS consists of five questions regarding changes in height (i.e. growth spurt), body hair (i.e. hair any place other than on the head, such as under the arms), skin (i.e. pimples), voice, and facial hair (males) or changes in height, body hair, skin, breast development, and menarche (females). For each question, caregivers and youth were asked to separately rate physical development on a 4-point scale (1 = has not begun yet, 2 = barely begun, 3 = definitely begun, 4 = seems complete), except for the menarche question, which consisted of a yes/no answer choice (yes = 4; no = 1). If menarche was reported, then follow-up questions were asked about the child’s menstrual cycle including age of first menstruation. An “I don’t know” option was also available for each item for both caregiver and youth as well as a “Refuse to answer” option on the youth self-report.

PDS values were utilized to calculate the following: average PDS score (41), adrenal- versus gonadal-related average PDS scores (42), and pubertal category score (43). These summary scores were computed separately for caregiver versus child report and separately for male and female participants. As previously described (42), gonadal PDS scores were created for females by averaging growth spurt, breast development, and menarche PDS items; for males, by averaging growth spurt, deepening of voice, and facial hair growth PDS items. Adrenal scores were created by averaging pubic/body hair and skin changes from PDS items for both males and females. The puberty category score was derived for males by summing the body hair growth, voice change, and facial hair items and categorizing them as follows: prepubertal = 3 (all one-point responses); early pubertal = 4 or 5 (no 3-point responses); midpubertal = 6–8 (no 4-point responses); late pubertal = 9–11; and, postpubertal = 12 (e.g. all 4-point responses) (43). The puberty category score was derived for females by summing the body hair growth and breast development and using the menarche variable for categorizing them as follows: prepubertal = 2 and no menarche; early pubertal = 3 and no menarche; midpubertal =>3 and no menarche; late pubertal <=7 and menarche; postpubertal = 8 and menarche (43).

For statistical purposes, items were considered as “missing” if the response was left blank and/or answered with “I don’t know” or “Refuse to answer”. In order to reduce over- or under-estimation of the pubertal outcome variables, participants were excluded if they had more than one “missing” value when calculating the average PDS score (**Supplemental Table 1**). For the more specific gonadal versus adrenal and pubertal category scores, individuals were excluded from being assigned these scores if any of the respective items were “missing”. Birth sex was utilized to classify participants as male or female, and gender identity was not incorporated into current analyses.

### Saliva Collection and Determination of Salivary Biomarkers

Given importance of assessing pubertal maturation with biomarkers, ABCD selected salivary sampling for assessment of 3 gonadal hormones given its noninvasive nature, ability to be collected without a phlebotomist, and ability to reliably reflect hormone levels. Salivary biomarkers utilized whole saliva collected *via* passive drool from each participating child with assistance from trained research assistants in the laboratory. The saliva collection was adapted from Granger and colleagues (44). Briefly, participants did not have any food, snacks, drinks, gum, candy, or mints in the 30 min prior to collection, no major meals in the 60 min prior to collection, and were asked to rinse their mouth with water 10 min prior to saliva collection to remove particulates. Collection times in the ABCD Study varied between 7:00 am and 7:00 pm. To protect the cold chain, samples were placed into a Nalgene Labtop Cooler [chilled before sampling in on-site freezer (-80° to -20°C) and kept inside a small lunchbox cooler] immediately after collection. Depending on the site, saliva samples were either placed inside an on-site freezer immediately after collection, while some sites placed the sample within a cooler placed inside a refrigerator (4°C) during neurocognitive testing, and then saliva samples were stored in an on-site freezer by the end of the testing day. Saliva samples were shipped 2–6 months after collection on dry ice to Salimetrics (Carlsbad, CA) and promptly assayed. All hormones were assayed in duplicate within a single day to avoid multiple freeze-thaw cycles (testosterone and DHEA in males and females, and 17-β estradiol in females only) using commercially available immunoassays specifically designed for use with saliva without modification to the manufacturers recommended protocol (Salimetrics). The following specifications are listed in order to reflect testosterone, DHEA, and estradiol: calibrator ranges (6.1–600 pg/ml; 10.2–1,000 pg/ml; 1–32 pg/ml, respectively); lower limits of sensitivity (1 pg/ml; 5 pg/ml; 0.1 pg/ml, respectively), incubation time (1.5 h; 3.5 h; 2.5 h, respectively) and correlation with serum (0.96; 0.86; 0.80, respectively).

**Figure 1:**
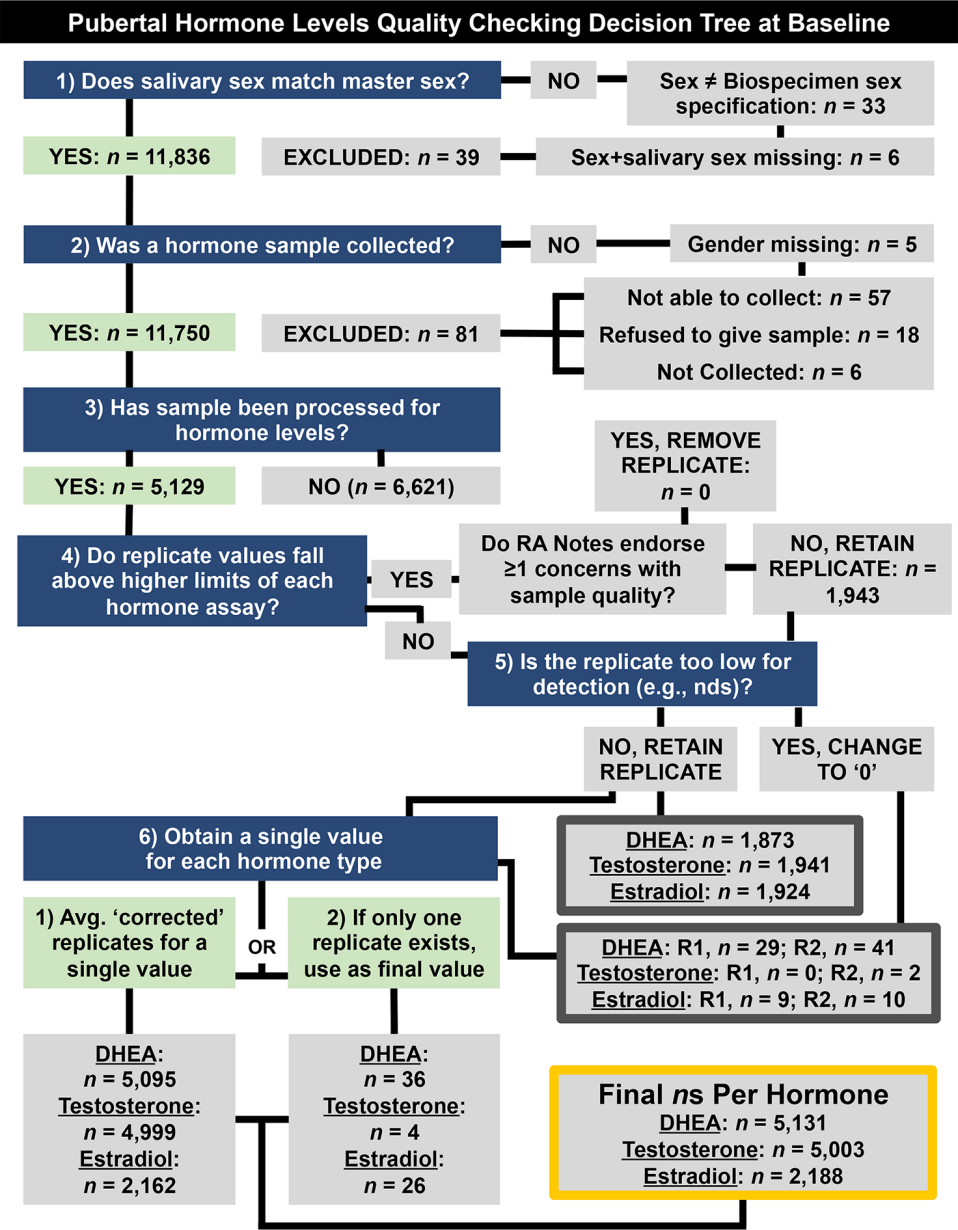
Decision tree for quality checking and generating a single hormone metric per participant at baseline (9–10 years old) (e.g., saliva assayed in duplicates, considerations for methodological concerns that may influence hormone level). Briefly, data were retained if (1) the sex specified during saliva collection matches sex at birth, as reported by parent participants, (2) if a salivary hormone sample was collected, and (3) if that sample had been processed. Replicate samples that fell below detection limits or were endorsed as problematic by research assistants (RAs) were not used to calculate participants’ hormone levels (4–6). NDS, not detectable sample; R1, Replicate 1. R2, Replicate 2.

To establish a single hormone value for each participant at baseline, a decision-tree was implemented for conducting quality control in each replicate value and to establish a final estimate of hormone level, as presented in **Figure 1**. To determine whether or not hormone levels varied as a function of methodological or physiological factors independent of pubertal maturation, we assessed relationships between hormone levels and the following independent factors relating to salivary sample collection: 1) collection time (minutes since midnight); 2) duration of salivary collection (minutes from start to finish of active collection); and, 3) time taken to place saliva sample into freezer for storage (minutes from collection finish time to placement into freezer on-site). The physiological factors included 1) caffeine intake in the past 12 hours (yes/no); and 2) vigorous physical exercise within the past 12 hours (yes/no), as recorded by the researcher from the child participant on the saliva collection day.

### Body Mass Index and Sociodemographic Variables

Anthropometric measurements of height and weight were taken as the average of up to three separate measures using professional grade equipment (e.g. physician weight beam scale with height rod). These data were then used to calculate body mass index *z*-scores (i.e., BMIz) and weight status (underweight: <5^th^ percentile; overweight: >85^th^ to <95^th^; obese: ≥ 95^th^ percentile) based on the individual’s age and biological sex using the SAS program provided by the 2000 CDC Growth Charts (ages 0 to 20 years) (45). ABCD’s Data Exploration and Analysis Portal (DEAP) variables were used for race and ethnicity, as well as socioeconomic factors. Specifically, race/ethnicity were combined to generate dummy codes with five dichotomous values, including White, Black, Hispanic, Asian, and Other/Multi-race. Family socioeconomic status (SES) was assessed *via* caregiver-reported total household income and highest household education. Household income was collected in bins (i.e. <$5,000, $5,000–$11,999, $12,000–$15,999, $16,000–$24,999, $25,000–$34,999, $35,000–$49,999, $50,000–$74,999, $75,000–$99,999, $100,000–$199,999, ≥$200,000). The DEAP variables for income were binned into a categorical factor representing low (<$50k), middle ($50–$100k) and high (≥$100k) income. Highest education was defined as the highest education attained among caregivers. It was reported in incremental categories ranging from never educated/kindergarten through doctoral-level graduate degree. The DEAP variable of highest education was used, representing categorical factors of less than high school (HS) education, HS education/GED, Some College (including Associate’s Degree), Bachelor’s Degree, and Postgraduate Degree.

### Latent Factors of Pubertal Maturation

While other studies have examined association with individual measures of hormonal and physical features (7, 16, 42, 46), we also applied a group factor analysis (GFA) to derive latent factors that encompassed integrated measures of both perceived physical features and hormone levels. Factor analysis is a useful tool for investigating constructs that are otherwise hard to directly measure but can be indirectly measured by observed variables (47). The goal of GFA is to identify latent factors that explain relationships within groups of variables from relationships seen between groups of variables (39). In the case of the current analyses, given the collection of 2 groups of variables with various dimensions (group 1: 5 response items from the PDS and group 2: 2 (or 3 in females) hormone levels measured from a salivary sample), the task is to identify factors that describe dependencies between the multiple groups of variables, while allowing for within-group factors that account for covariance unique to each group. The GFA solution differs from canonical correlation analysis or standard exploratory factor analysis by utilizing a Bayesian inferential framework to place an Automatic Relevance Determination prior on the factor solution, which assumes a low-rank representation of the factor loading (48). The main advantages of GFA are that it is conceptually simple, as it differentiates within-group from between-group associations, and it allows for factor analysis in scenarios with two or more groups of data, giving factor solutions that are not merely accounted for due to method variance resulting from one variable grouping. The GFA solution comprises a set of factors that contain a projection vector for each of the variable groups having non-zero weights for that factor (39). Given that hormone levels and physical features are dependent on biological sex, GFAs were implemented separately for males and females. Latent factors accounting for more than 10% of the variance were chosen. To ensure factors were robust and to further test the stability of the factors for PDS and hormones, we performed the following: (1) GFA analyses were replicated ten times (averaged factor loadings are presented); 2) split-half samples were randomly generated to test replication in two separate sub-samples; and, 3) GFA implementations were subsequently extended to include sociodemographic measures by including BMIz, then BMIz + SES (parental education and family income), then pre-residualized BMIz + SES + race/ethnicity (Black, Hispanic, Asian, White, Other). For completeness, in addition to PDS items reported by caregivers, we also performed a GFA using PDS values from youth self-report.

### Analytic Strategy

Statistical evaluation of the data was performed in R (version 3.6). Group Factor Analyses [GFA (39, 49)] were conducted using the R package GFA (50). Mixed-effects model analyses were conducted using the R package nlme and/or gamm4 (51, 52). All mixed model and GFA analyses were conducted with complete cases only. Data are presented as mean, standard deviation, and frequencies. Caregiver-youth agreement for PDS items was examined using a polychoric correlation coefficient [rho (53)] and a weighted Cohen’s kappa coefficient [k (54)]. The polychoric correlation is useful for examining agreement between ordered category data (53), whereas k is weighted to consider random chance of agreement (54). Exploratory analyses were also performed to examine if agreement varied as a function of the reporting caregiver (i.e. biological mother versus biological father).

Separate mixed-effects models were performed for summary scores of physical maturation and hormone levels to examine the association of sociodemographic variables in relation to markers of pubertal status. The sociodemographic variables were selected *a priori*, which have all been shown to relate to puberty, based on past literature (16–19, 23, 55). Variables included a main effect and potential sex difference (i.e. interaction term) for age (in months), race/ethnicity, highest household education obtained, and household income. For the effect of age and age-by-sex interactions, we also explored both linear and non-linear associations using the linear mixed effect modeling (LME; R package nlme) and general additive modeling (GAM; gamm4), respectively. Given the study design and nested data structure, all models included random intercepts for ABCD site and family relationship for each participant as to account for between-site variability and within-family correlations. In these models, the reference groups for the categorical variables were age = 108 months, sex = male, ethnicity = Hispanic, parental education = high school diploma/GED, total family income = middle ($50k–$100k), and weight status = healthy weight. It is important to note that the results do not differ as a function of changing the reference group. In models for hormone levels, we also included important methodological and physiological factors that may affect salivary hormone levels, including caffeine intake (yes/no), physically active (yes/no), time of collection since midnight (minutes), collection duration (minutes), and time from collection to freeze (minutes).

## Results

### Self-Reported Physical Markers of Perceived Puberty

**Figure 2:**
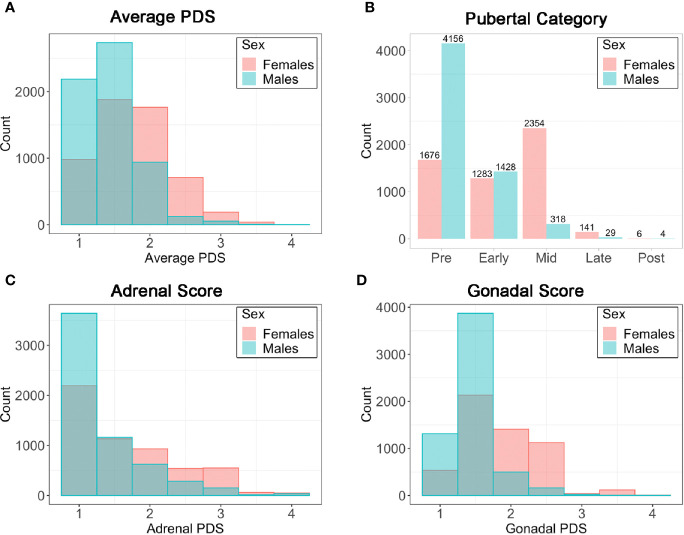
Frequencies (N) for caregiver summary scores from the Pubertal Development Scale (PDS). **(A)** Average PDS score ranging from 1=not begun to 4=complete; **(B)** Pubertal Category score ranging from pre- to post- pubertal; **(C)** Adrenal score averaging adrenal PDS items and ranging from 1=not begun to 4=complete; **(D)** Gonadal score averaging gonadal PDS items and ranging from 1=not begun to 4=complete.

Item scores for caregiver and youth reports are presented in **Table 1**. In females, the caregiver reporting was primarily the biological mothers (86%) [biological fathers (8.9%), adoptive parents (2.6%), custodial parent (1%), or other (1.5%)]. Similarly, the caregiver reporting for males was primarily biological mothers (85%) [biological fathers (11%), adoptive parent (2.6%), custodial parent (0.9%), or other (1.2%)]. Only a small percentage of youth (≤1%) refused to answer any given item (**Table 1**). A larger percentage of both males and females reported “I don’t know” as compared to caregivers, especially for Item 1 asking about growth in height (**Table 1**). **Figure 2** presents the frequencies and distributions of summary scores based on caregiver reports, and caregiver and youth reports for summary scores are presented in **Supplemental Table 1**. Based on the caregiver’s report, overall average PDS scores, gonadal, and adrenal scores were higher (more mature) in females as compared to males. Based on caregivers, 70.0% of males and 30.7% of females were perceived as prepubertal, 24.1% of males and 23.5% of females were perceived as being in the early-pubertal stage, and 5.3% of males and 43.0% of females were reported to be in the mid-pubertal range. Youth, however, reported more mature levels of perceived physical development as compared to their caregivers, and male and female youth reported similar overall PDS averages, gonadal, and adrenal scores. Based on youth self-report, 29.9% of males and 25.6% of females identified as being in the prepubertal stage, 47.62% of males and 26.84% of females reported being in the early-pubertal stage, and 20.5% of males and 44.2% of females identified as mid-pubertal stage.

**Table 1:** Frequencies of scores reported on each item of the Pubertal Development Scale based on caregiver and youth report for each sex.

	Caregiver Report		Youth Report	
	Males	Females	Males	Females
	N = 6,141	N = 5,659	N = 6,158	N = 5,628
**Height**				
Not Begun	22% (N = 1,381)	14% (N = 791)	14% (N = 844)	11% (N = 593)
Barely Started	20% (N = 1,231)	16% (N = 896)	20% (N = 1,246)	19%, (N = 1,048)
Underway	51% (N = 3,146)	63% (N = 3,543)	25% (N = 1,570)	22% (N = 1,257)
Complete	3% (N = 167)	4% (N = 208)	6% (N = 392)	6% (N = 362)
I don’t know	4% (N = 216)	4% (N = 221)	34% (N = 2,082)	42% (N = 2,339)
Refuse to answer	–	–	<1% (N = 23)	<1% (N = 28)
				
**Body Hair**				
Not Begun	72% (N = 4,427)	52% (N = 2,922)	49% (N = 3,022)	46% (N = 2,578)
Barely Started	15% (N = 942)	20% (N = 1,153)	30% (N = 1,876)	30% (N = 1,691)
Underway	8% (N = 499)	23% (N = 1,277)	10% (N = 640)	12% (N = 690)
Complete	2% (N = 119)	3% (N = 160)	5% (N = 318)	5% (N = 309)
I don’t know	3% (N = 154)	3% (N = 147)	4% (N = 277)	6% (N = 324)
Refuse to answer	–	–	<1% (N = 24)	1% (N = 34)
				
**Skin Changes**				
Not Begun	73% (N = 4,492)	56% (N = 3,168)	50% (N = 3,098)	44% (N = 2,474)
Barely Started	18% (N = 1,116)	27% (N = 1,519)	25% (N = 1,569)	31% (N = 1,759)
Underway	6% (N = 358)	14% (N = 809)	7% (N = 435)	9% (N = 529)
Complete	1% (N = 62)	1% (N = 75)	3% (N = 157)	2% (N = 132)
I don’t know	2% (N = 113)	2% (N = 88)	14% (N = 873)	13% (N = 716)
Refuse to answer	–	–	<1% (N = 25)	<1% (N = 18)
				
**Facial Hair**				
Not Begun	91% (N = 5,595)	–	53% (N = 3,273)	–
Barely Started	6% (N = 387)	–	30% (N = 1,874)	–
Underway	2% (N = 93)	–	8% (N = 493)	–
Complete	<1% (N = 16)	–	3% (N = 187)	–
I don’t know	1% (N = 50)	–	5% (N = 320)	–
Refuse to answer	–	–	<1% (N = 10)	–
				
**Voice Change**				
Not Begun	93% (N = 5,699)	–	75% (N = 4,630)	–
Barely Started	5% (N = 309)	–	17% (N = 1,041)	–
Underway	1% (N = 63)	–	3% (N = 157)	–
Complete	<1% (N = 10)	–	1% (N = 38)	–
I don’t know	1% (N = 60)	–	4% (N = 273)	–
Refuse to answer	–	–	<1% (N = 18)	–
				
**Breast Development**				
Not Begun	–	39% (N = 2,208)	–	31% (N = 1,736)
Barely Started	–	35% (N = 1,973)	–	35% (N = 1,977)
Underway	–	24% (N = 1,372)	–	13% (N = 755)
Complete	–	1% (N = 48)	–	2% (N = 126)
I don’t know	–	1% (N = 57)	–	17% (N = 959)
Refuse to answer	–	–	–	1% (N = 68)
				
**Menarche**				
Yes	–	3% (N = 154)	–	3% (N = 163)
No	–	96% (N = 5,455)	–	87% (N = 4,871)
I don’t know	–	1% (N = 49)	–	10% (N = 537)
Refuse to answer	–	–	–	1% (N = 55)
				
**Menarche Age** (years)	–		–	
7		<1% (N = 1)		1.8 (N = 3)
8		5.8% (N = 9)		7.4% (N = 12)
9		29.2% (N = 45)		31.3% (N = 51)
10		61.7% (N = 95)		53.4% (N = 87)
I don’t know		2.6% (N = 4)		5.5% (N = 9)
Refuse to answer		–		6.1% (N = 1)

When examining the agreement between caregiver versus youth report on the PDS (**Table 2**), rho values ranged from 0.18 to 0.38 for males and 0.27 to 0.98 for females. Kappa values which consider possible agreement due to chance, ranged from 0.05 to 0.20 for males and 0.22 to 0.81 in females. Agreement statistics were similar regardless of which caregiver completed the PDS (biological father or mother) for males or females (**Supplemental Table 2**). Given the previous literature showing that youth tend to over-report their perceived physical development at earlier ages (56), caregiver PDS scores were utilized in examining perceived physical changes and hormonal features in all further analyses.

**Table 2:** Caregiver-youth agreement for Pubertal Development Scale items and summary scores by sex.

PDS Items	Males			Females		
	N	Kappa (95% CI)	Rho (SE)	N	Kappa (95% CI)	Rho (SE)
Height	3,893	0.155 (0.12, 0.18)	0.18 (0.02)	3,122	0.22 (0.19, 0.25)	0.28 (0.02)
Body Hair	5,683	0.13 (0.10, 0.15)	0.21 (0.02)	5,120	0.37 (0.35, 0.40)	0.47 (0.01)
Skin Changes	5,125	0.195 (0.17, 0.22)	0.32 (0.02)	4,807	0.37 (0.34, 0.40)	0.46 (0.02)
Voice Change	5,770	0.143 (0.08, 0.12)	0.29 (0.02)	4,541	0.52 (0.49, 0.54)	0.62 (0.01)
Facial Hair	5,743	0.143 (0.11, 0.17)	0.38 (0.03)	4,980	0.81 (0.76, 0.86)	0.98 (0.01)
**PDS Summary Scores**	**N**	**Kappa (95% CI)**	**Rho (SE)**	**N**	**Kappa (95% CI)**	**Rho (SE)**
Average PDS	5,176	0.20 (0.17, 0.22)	0.23 (0.01)	4,229	0.53 (0.51, 0.56)	0.53 (0.01)
Gonadal Score	3,643	0.18 (0.15, 0.21)	0.22 (0.02)	2,501	0.55 (0.52, 0.59)	0.55 (0.01)
Adrenal Score	4,844	0.18 (0.15, 0.21)	0.26 (0.02)	4,491	0.44 (0.41, 0.47)	0.50 (0.01)
PDS Category	5,261	0.05 (0.03, 0.07)	0.25 (0.02)	3,936	0.22 (0.18, 0.26)	0.67 (0.01)

Kappa coefficient means (95% confidence intervals, CI) as well as polychoric coefficients rho (standard error, SE).

### Salivary Hormones

Mean hormone levels for males and females and methodological covariates are presented in **Table 3**. No statistical hormone outliers were observed. Despite a rather narrow age range, age-related increases were seen in each hormone for both males and females (**Supplemental Figures 1–3**).

**Table 3:** Summary of hormone levels and salivary collection covariates in females and males.

Hormone Levels	Males			Females		
	N	Mean ± SD	IQR	N	Mean ± SD	IQR
DHEA (pg/ml)	2,676	57.78 ± 43.78	50.04	2,430	76.87 ± 56.28	63.15
Testosterone (pg/ml)	2,622	32.80 ± 17.70	21.41	2,358	37.07 ± 18.37	22.94
Estradiol (pg/ml)	–	--	–	2,168	1.17 ± 0.52	0.69
**Covariates**	**N**	**Mean ± SD**	**IQR**	**N**	**Mean ± SD**	**IQR**
Caffeine in past 12 hours (N)	2,376	Yes = 161/No = 2,210	–	1,707	Yes = 95/No = 1,612	–
Physical activity in past 12 hours (N)	2,366	Yes = 318/No = 2,048	–	1,703	Yes = 216/No = 1,487	–
Time Since Midnight (minutes)	2,381	777.58 ± 181.73	313	1,712	776.53 ± 177.18	311
Collection Duration (minutes)	2,318	6.81 ± 5.44	6	1,676	7.49 ± 5.89	5
Time to Freeze (minutes)	2,335	2.69 ± 12.31	1	1,686	3.05 ± 14.38	1

Mean and SD and Interquartile Range (IQR) unless otherwise noted.

### Associations Between Physical and Hormonal Features

**Figure 3:**
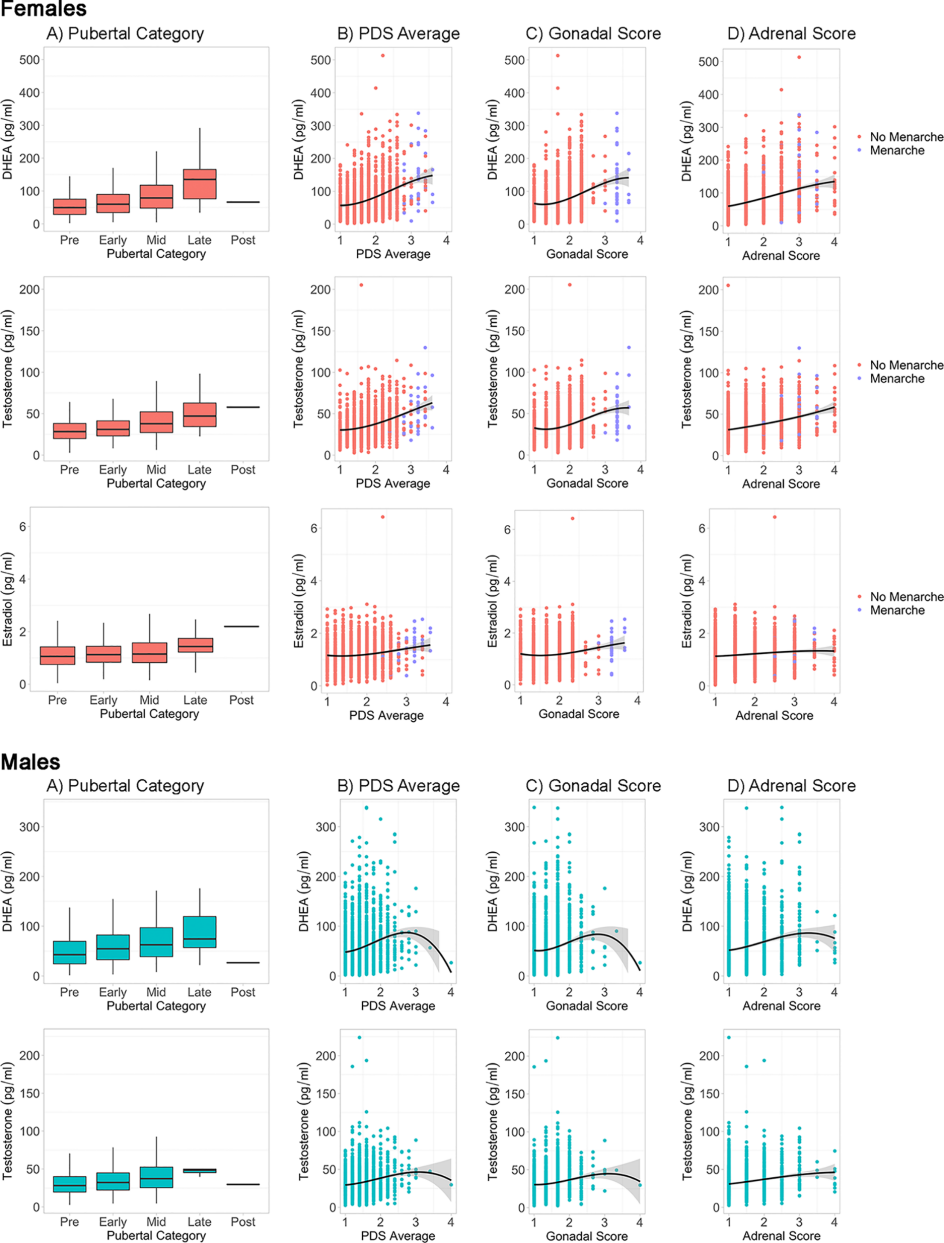
Caregiver based Pubertal Development Scale (PDS) summary scores and hormone levels by sex. For each sex, hormone levels are plotted by PDS summary scores, including **(A)** Pubertal Status Category, **(B)** Average PDS of all items, **(C)** Gonadal Score of PDS, and **(D)** Adrenal Score of PDS. Line represents cubic spline function of the data. Females are plotted by menarche status.

In the sub-sample of youth with both hormone levels and caregiver PDS, hormone levels are shown by perceived pubertal category for females and males (**Figure 3A**, **STable 3**). As expected, mean levels of DHEA (females: n = 1,713, F(4, 1,708) = 45.4, p < 0.0000001; males: n = 2,383, F(4, 2,378) = 15.69, p < 0.0000001), testosterone (females: n = 1,713, F(4, 1,708) = 36.84, p < 0.0000001; males: n = 2,383, F(4, 2,378) = 15.53, p < 0.0000001), and estradiol (females: n = 1,733, F(4, 1,708) = 7.7, p < 0.0001) were systematically higher for subsequent pubertal categories, especially from the prepubertal to late pubertal stages. Using the PDS average (**Figure 3B**), Gonadal score (**Figure 3C**), and Adrenal score (**Figure 3D**), similar patterns of higher hormone levels with more advanced perceived physical development across the prepubertal to late pubertal stages, albeit with the exception of extremely large variance seen at the most advanced physical stages for males given the few subjects within these late stage categories. Correlations between PDS summary scores and hormone levels ranged from 0.13 to 0.19 in males and 0.10 to 0.34 in females (**Supplemental Table 3**).

### Sociodemographic Associations

**Figure 4:**
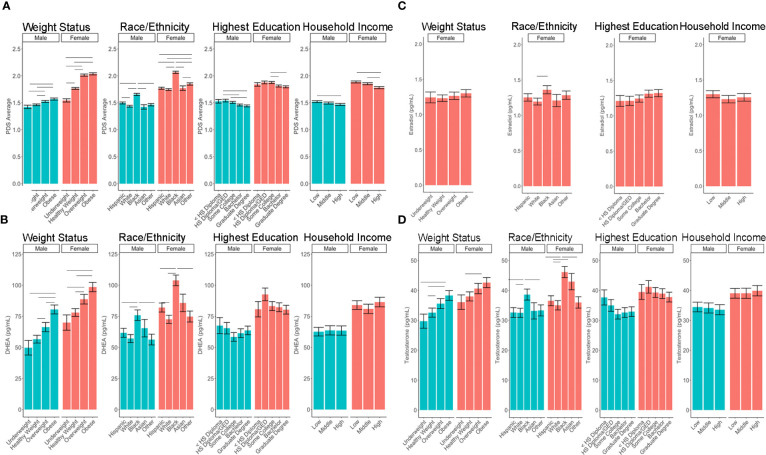
Post-hoc comparisons of sex differences in the associations between sociodemographic measures and pubertal outcomes of **(A)** PDS Average, **(B)** DHEA, **(C)** Estradiol, **(D)** Testosterone. Means and standard error (SE) for the fixed effects of weight status, race/ethnicity, highest parental education, and household income by sex, while adjusting for means of all other variables in the model. Lines denote p <0.05 using Tukey multiple comparison correction. HS, High school; GED, General Education Development. Income binned into a numerically coded factor representing low (<$50k), middle ($50–$100k), and high (≥$100k) income.

A summary of sociodemographic characteristics by perceived pubertal stage are reported in **Table 4**. Mixed-effects models to examine the fixed effects for sex, age (in months), weight status, race/ethnicity, parental education, and household income for each PDS summary score and salivary hormone are presented in **Tables 5** and **6**. The fixed effects accounted for more variance in physical scores (Marginal *R* ^2^: 0.21 to 0.32) as compared to hormone levels (Marginal *R* ^2^: 0.10 to 0.21). Associations between age and physical features were found to be linear in both sexes. In contrast, associations between age and androgen levels were non-linear in females, but linear in males; suggesting larger age-related increases in DHEA and testosterone in females at age 10 than age 9. In contrast, estradiol levels were found to be associated in a linear fashion with age. Adjusting for all sociodemographic characteristics in the same model (i.e. age, sex, weight status, race/ethnicity, highest education, and household income), results also showed more advanced pubertal maturation as indexed by either self-report of more advanced physical features (**Table 5**) or higher androgen levels (**Table 6**) for 1) females versus males, 2) overweight versus healthy weight youth, and 3) those self-identifying as Black as compared to all other race/ethnicity categories (**Figure 4**, **Supplemental Tables 4–7**). Effects of age, body weight status, and race/ethnicity on physical and hormone outcomes were also larger in females as compared to males (**Figure 4**, **Supplemental Tables 4–7**). Lastly, associations between highest household education and household income were only seen for self-report measures of physical maturation, with higher education associated with less physical maturation in both males and females and higher household income associated with less physical maturation in females (**Table 5**, **Supplemental Tables 4–7**).

**Table 4:** Summary of sociodemographic variables for each Pubertal Development Scale derived categorical stage of perceived puberty based on caregiver report for females and males.

	Male					Female				
	Pre	Early	Mid	Late	Post	Pre	Early	Mid	Late	Post
**N**	4,140	1,422	314	29	4	1,664	1,278	2,328	138	6
**Age** (in months)	118.5 ± 7.43	120.5 ± 7.37	120.4 ± 7.25	122.1 ± 7.09	122.8 ± 8.18	115.9 ± 6.67	118.7 ± 7.39	120.5 ± 7.30	125.3 ± 5.37	126.8 ± 3.25
**Weight Status**										
Healthy Weight	73.8%	21.6%	4.2%	0.3%	0.1%	38.4%	24.3%	35.9%	1.4%	0.0%
Underweight	79.4%	18.6%	2.0%	0.0%	0.0%	58.5%	21.4%	20.1%	0.0%	0.0%
Overweight	65.2%	27.7%	6.2%	0.9%	0.0%	13.6%	25.0%	55.8%	5.5%	0.1%
Obese	58.3%	31.2%	9.4%	1.0%	0.1%	8.1%	20.1%	66.2%	5.0%	0.6%
**Race/Ethinicity**										
Hispanic	64.6%	26.8%	7.9%	0.7%	0.1%	26.7%	26.8%	42.0%	4.3%	0.2%
White	78.9%	19.4%	1.6%	0.1%	0.0%	38.4%	26.5%	34.3%	0.9%	0.0%
Black	41.4%	39.6%	16.7%	2.2%	0.1%	10.4%	9.8%	73.5%	5.8%	0.5%
Asian	73.7%	23.7%	2.6%	0.0%	0.0%	36.6%	32.5%	29.3%	1.6%	0.0%
Other	70.3%	23.2%	6.0%	0.2%	0.3%	27.9%	21.6%	47.8%	2.6%	0.0%
**Highest Education**										
< HS Diploma	57.7%	28.0%	11.0%	2.8%	0.4%	18.1%	24.3%	52.1%	5.0%	0.4%
HS Diploma/GED	55.7%	27.4%	15.0%	1.6%	0.4%	17.9%	18.3%	58.0%	5.6%	0.2%
Some College	62.8%	28.1%	8.5%	0.5%	0.1%	22.3%	19.2%	54.6%	3.6%	0.2%
Bachelor	74.2%	22.6%	2.9%	0.2%	0.0%	34.7%	26.3%	37.0%	1.9%	0.1%
Post Graduate Degree	77.4%	20.7%	1.8%	0.1%	0.0%	38.9%	26.2%	33.9%	1.0%	0.0%
**Family Income**										
<50K	58.5%	30.4%	9.9%	1.1%	0.1%	18.9%	19.5%	56.3%	4.9%	0.3%
≥50K & <100K	71.7%	23.1%	4.6%	0.5%	0.1%	31.8%	23.7%	42.2%	2.3%	0.0%
≥100K	78.0%	20.1%	1.9%	0.0%	0.0%	39.5%	26.7%	32.8%	1.1%	0.0%

Mean and SD for age, and percentage of each categorical outcome across the 5 pubertal stages.

**Table 5:** Sociodemographic differences for Pubertal Development Scale summary scores.

Main Effects	A) PDS Average					B) Gonadal Score					C) Adrenal Score				
	*b*	* *	CI		p	*b*	CI			p	*b*		CI		p
**Female (vs. Male)**	0.07	-0.01	–	0.14	0.08	0.07	-0.004	–	0.14	0.06	0.03	-0.08	–	0.14	0.61
**Interview age (months)**	0.01	0.005	–	0.01	**<0.001**	0.004	0.002	–	0.005	**<0.001**	0.01	0.01	–	0.01	**<0.001**
**Underweight (vs. Healthy Weight)**	-0.04	-0.09	–	0.02	0.18	-0.04	-0.10	–	0.01	0.14	-0.04	-0.12	–	0.05	0.37
**Overweight (vs. Healthy Weight)**	0.06	0.03	–	0.09	**<0.001**	0.06	0.03	–	0.09	**<0.001**	0.05	0.01	–	0.10	**0.02**
**Obese (vs. Healthy Weight)**	0.11	0.08	–	0.14	**<0.001**	0.09	0.06	–	0.12	**<0.001**	0.14	0.10	–	0.19	**<0.001**
**White (vs. Hispanic)**	-0.06	-0.09	–	-0.03	**<0.001**	-0.07	-0.11	–	-0.04	**<0.001**	-0.04	-0.09	–	0.01	0.08
**Black (vs. Hispanic)**	0.16	0.12	–	0.20	**<0.001**	0.09	0.05	–	0.13	**<0.001**	0.27	0.21	–	0.33	**<0.001**
**Asian (vs. Hispanic)**	-0.07	-0.15	–	0.01	0.08	-0.10	-0.18	–	-0.02	**0.02**	-0.02	-0.15	–	0.10	0.70
**Other (vs. Hispanic)**	-0.03	-0.07	–	0.01	0.19	-0.05	-0.09	–	-0.01	**0.02**	0.01	-0.06	–	0.07	0.86
**< HS Diploma (vs. HS Diploma/GED)**	-0.02	-0.09	–	0.04	0.49	0.02	-0.05	–	0.08	0.62	-0.11	-0.21	–	-0.003	**0.04**
**Some College (vs. HS Diploma/GED)**	-0.04	-0.08	–	0.01	0.10	-0.04	-0.08	–	0.002	0.06	-0.05	-0.12	–	0.02	0.15
**Bachelor’s (vs. HS Diploma/GED)**	-0.09	-0.14	–	-0.04	**<0.001**	-0.09	-0.14	–	-0.05	**<0.001**	-0.11	-0.18	–	-0.03	**0.004**
**Post Graduate Degree (vs. HS Diploma/GED)**	-0.10	-0.15	–	-0.06	**<0.001**	-0.12	-0.16	–	-0.07	**<0.001**	-0.12	-0.20	–	-0.05	**0.001**
**Household Income <50K (vs. ≥50K & <100K)**	0.02	-0.01	–	0.06	0.14	0.01	-0.02	–	0.05	0.40	0.04	-0.01	–	0.09	0.09
**Household Income ≥100K (vs. ≥50K & <100K)**	-0.02	-0.05	–	0.004	0.09	-0.02	-0.05	–	0.01	0.11	-0.02	-0.06	–	0.02	0.30
**Interactions**	***b***	*** ***	**CI**	** **	**p**	***b***	**CI**			**p**	***b***	*** ***	**CI**	** **	**p**
**Female : Interview age (months)**	0.02	0.01	–	0.02	**<0.001**	0.01	0.01	–	0.02	**<0.001**	0.02	0.01	–	0.02	**<0.001**
**Female : Underweight (vs. Healthy Weight)**	-0.18	-0.26	–	-0.10	**<0.001**	-0.17	-0.25	–	-0.10	**<0.001**	-0.21	-0.32	–	-0.09	**<0.001**
**Female : Overweight (vs. Healthy Weight)**	0.18	0.14	–	0.22	**<0.001**	0.18	0.14	–	0.23	**<0.001**	0.18	0.12	–	0.24	**<0.001**
**Female : Obese (vs. Healthy Weight)**	0.16	0.11	–	0.20	**<0.001**	0.21	0.16	–	0.25	**<0.001**	0.09	0.02	–	0.15	**0.01**
**Female : White (vs. Hispanic)**	0.04	-0.01	–	0.08	0.12	0.03	-0.01	–	0.08	0.15	0.05	-0.02	–	0.11	0.17
**Female : Black (vs. Hispanic)**	0.14	0.08	–	0.19	**<0.001**	0.11	0.06	–	0.16	**<0.001**	0.18	0.10	–	0.27	**<0.001**
**Female : Asian (vs. Hispanic)**	0.07	-0.04	–	0.18	0.23	0.11	-0.001	–	0.22	0.05	-0.04	-0.21	–	0.14	0.69
**Female : Other (vs. Hispanic)**	0.10	0.04	–	0.16	**0.001**	0.08	0.02	–	0.14	**0.01**	0.13	0.04	–	0.22	**0.003**
**Female:<HS Diploma (vs. HS Diploma/GED)**	-0.01	-0.10	–	0.08	0.83	0.00	-0.09	–	0.09	1.00	0.06	-0.08	–	0.20	0.40
**Female : Some College (vs. HS Diploma/GED)**	0.03	-0.03	–	0.10	0.27	0.02	-0.04	–	0.08	0.51	0.10	0.00	–	0.19	**0.04**
**Female : Bachelor’s (vs. HS Diploma/GED)**	0.02	-0.05	–	0.09	0.53	0.04	-0.03	–	0.10	0.30	0.04	-0.06	–	0.14	0.45
**Female : Post Graduate Degree (vs. HS Diploma/GED)**	0.02	-0.05	–	0.09	0.52	0.04	-0.03	–	0.11	0.27	0.05	-0.06	–	0.15	0.38
**Female : Household Income <50K (vs. ≥50K & <100K)**	0.01	-0.04	–	0.05	0.83	0.02	-0.03	–	0.06	0.45	-0.02	-0.09	–	0.05	0.63
**Female : Household Income ≥100K (vs. ≥50K & <100K)**	-0.05	-0.09	–	-0.01	**0.02**	-0.05	-0.09	–	-0.02	**0.01**	-0.05	-0.11	–	0.01	0.11
Observations	10,424					10,293					10409				
Marginal R^2^	0.32					0.30					0.21				
Conditional R^2^	0.65					0.59					0.63				

Each column reflects models including all variables by which the dependent variable is A) PDS Average, B) Gonadal Score C) Adrenal Score. Unstandardized beta (b) coefficients, confidence intervals (CI), and p-values for each fixed effect in the model. Bold denotes significant values for p <0.05. Abbreviations: HS, High school; GED, General Education Development. Income binned into a numerically coded factor representing low (<$50k), middle ($50–$100k), and high (≥$100k) income.

**Table 6:** Sociodemographic differences in hormone levels.

Smoothed Terms	A) DHEA (pg/mL)					B) Testosterone (pg/mL)					C) Estradiol (pg/mL; females only)				
	*edf*				p	*edf*				p	edf				p
s(age): Males	1.0				**<0.001**	1.0				**<0.001**	--				--
s(age): Females	1.74				**<0.001**	1.33				**<0.001**	1.0				**<0.001**
**Main Effects**	***b***	*** ***	**CI**	*** ***	**p**	***b***	*** ***	**CI**	*** ***	**p**	**b**	** **	**CI**	** **	**p**
Female (vs. Male)	25.87	12.636	–	39.101	**<0.001**	4.54	-0.222	–	9.291	0.06	--	--			--
Underweight (vs. Healthy Weight)	-6.56	-16.915	–	3.792	0.21	-2.86	-6.599	–	0.884	0.13	0.01	-0.115	–	0.136	0.87
Overweight (vs. Healthy Weight)	9.87	4.487	–	15.25	**<0.001**	3.00	1.077	–	4.931	**0.002**	0.03	-0.033	–	0.100	0.32
Obese (vs. Healthy Weight)	24.39	19.014	–	29.774	**<0.001**	5.60	3.655	–	7.534	**<0.001**	0.07	0.002	–	0.136	**0.04**
White (vs. Hispanic)	-4.78	-10.791	–	1.230	0.12	-0.09	-2.283	–	2.102	0.94	-0.06	-0.134	–	0.017	0.13
Black (vs. Hispanic)	14.93	7.044	–	22.81	**<0.001**	5.99	3.081	–	8.898	**<0.001**	0.11	0.013	–	0.203	**0.03**
Asian (vs. Hispanic)	3.64	-9.824	–	17.103	0.60	0.56	-4.300	–	5.422	0.82	-0.04	-0.202	–	0.115	0.59
Other (vs. Hispanic)	-5.50	-13.522	–	2.526	0.18	0.50	-2.428	–	3.425	0.74	0.03	-0.065	–	0.129	0.52
<HS Diploma (vs. HS Diploma/GED)	1.78	-10.753	–	14.311	0.78	2.67	-1.850	–	7.180	0.25	0.00	-0.146	–	0.143	0.98
Some College (vs. HS Diploma/GED)	-7.13	-15.085	–	0.823	0.08	-2.92	-5.797	–	-0.045	**0.05**	0.03	-0.069	–	0.134	0.53
Bachelor's (vs. HS Diploma/GED)	-3.89	-12.577	–	4.803	0.38	-2.37	-5.512	–	0.779	0.14	0.10	-0.008	–	0.210	0.07
Post Graduate Degree (vs. HS Diploma/GED)	-1.78	-10.626	–	7.072	0.69	-2.12	-5.308	–	1.074	0.19	0.11	-0.003	–	0.222	0.06
Household Income <50K (vs. ≥50K & <100K)	-1.70	-7.657	–	4.253	0.58	0.33	-1.833	–	2.486	0.77	0.07	-0.001	–	0.141	**0.05**
Household Income ≥100K (vs. ≥50K & <100K)	-0.66	-5.747	–	4.423	0.80	-0.64	-2.480	–	1.209	0.50	0.03	-0.035	–	0.086	0.41
**Interactions**	***b***	**CI**			**p**	***b***	**CI**			**p**	**b**	**CI**			**p**
Female: Underweight (vs. Healthy Weight)	-1.52	-16.552	–	13.517	0.84	0.97	-4.421	–	6.360	0.72	--	--			--
Female: Overweight (vs. Healthy Weight)	1.07	-6.811	–	8.940	0.79	-0.39	-3.221	–	2.443	0.79	--	--			--
Female: Obese (vs. Healthy Weight)	-3.21	-11.105	–	4.677	0.43	-0.98	-3.823	–	1.873	0.50	--	--			--
Female: White (vs. Hispanic)	-5.22	-13.241	–	2.799	0.20	-1.40	-4.292	–	1.485	0.34	--	--			--
Female: Black (vs. Hispanic)	6.09	-4.494	–	16.671	0.26	3.63	-0.192	–	7.459	0.06	--	--			--
Female: Asian (vs. Hispanic)	0.01	-18.638	–	18.664	1.00	5.82	-0.842	–	12.481	0.09	--	--			--
Female: Other (vs. Hispanic)	-2.34	-13.409	–	8.729	0.68	-1.06	-5.060	–	2.939	0.60	--	--			--
Female: <HS Diploma (vs. HS Diploma/GED)	-15.11	-32.626	–	2.404	0.09	-4.35	-10.695	–	1.998	0.18	--	--			--
Female: Some College (vs. HS Diploma/GED)	-3.47	-15.171	–	8.240	0.56	1.04	-3.173	–	5.247	0.63	--	--			--
Female: Bachelor's (vs. HS Diploma/GED)	-8.02	-20.662	–	4.621	0.21	0.11	-4.454	–	4.676	0.96	--	--			--
Female: Post Graduate Degree (vs. HS Diploma/GED)	-12.18	-25.105	–	0.741	0.07	-1.29	-5.950	–	3.361	0.59	--	--			--
Female: Household Income <50K (vs. ≥50K & <100K)	4.47	-3.928	–	12.86	0.30	-0.32	-3.362	–	2.716	0.84	--	--			--
Female: Household Income ≥100K (vs. ≥50K & <100K)	6.70	-0.463	–	13.858	0.07	1.48	-1.111	–	4.067	0.26	--	--			--
**Covariates**	***b***	**CI**			**p**	***b***	**CI**			**p**	**b**	**CI**			**p**
Caffeine in Past 12 hours (Yes)	-3.09	-8.913	–	2.741	0.30	-1.04	-3.142	–	1.058	0.33	0.11	0.01	–	0.21	**0.03**
Physically active in Past 12 hours (Yes)	0.58	-3.761	–	4.914	0.79	-1.14	-2.699	–	0.410	0.15	0.07	-0.002	–	0.141	0.06
Since Midnight in minutes	-0.01	-0.017	–	0.001	0.08	-0.003	-0.007	–	-0.0002	**0.04**	0.0003	0.0002	–	0.0005	**<0.001**
Collection Duration in minutes	0.61	0.357	–	0.866	**<0.001**	0.25	0.160	–	0.342	**<0.001**	0.01	0.003	–	0.011	**0.001**
Time to Freeze sample in minutes	-0.02	-0.129	–	0.100	0.80	-0.02	-0.065	–	0.017	0.25	0.00	-0.002	–	0.001	0.64
Observations	4455					4349					1908				
Adjusted R^2^		0.21					0.18					0.10			

Each column reflects models including all variables by which the dependent variable is A) DHEA, B) Testosterone, C) Estradiol. Effective Degrees of Freedom (edf) for smoothed effects of age and unstandardized beta (b) coefficients, confidence intervals (CI), and p-values for each fixed effect in the model. Bold denotes significant values for p <0.05. Abbreviations: HS, High school; GED, General Education Development. Income binned into a numerically coded factor representing low (<$50k), middle ($50–$100k) and high (≥$100k) income.

### Latent Variables for Physical and Hormonal Features

**Figure 5:**
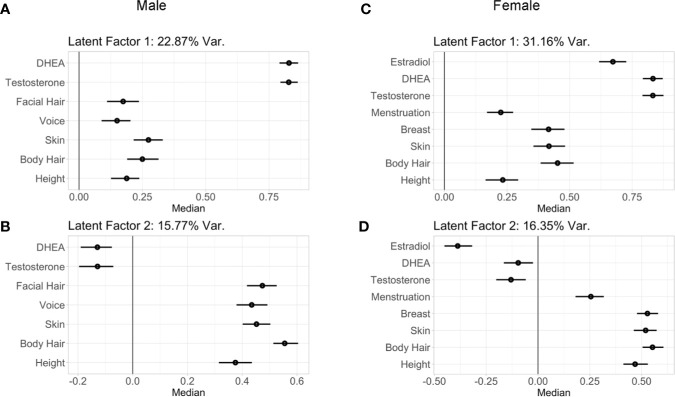
Latent factor loadings (median and 95% confidence intervals) of each predictor as identified by the two robust components of the group factor analyses. These analyses examined within and between variance in both perceived physical changes from the PDS as well as hormone levels in males **(A, B)** and females **(C, D)**. These two latent factors capture the wide range of individual variability seen between physical and hormone metrics of early puberty among children, with latent factor 1 driven by hormone levels, and the latent factor 2 driven by physical maturation. Latent factor 1 accounted for 22.87% of variance of the pubertal measurements in **(A)** males and 31.16% in females **(C)**. Latent factor 2 accounted for 15.77% of variance of the pubertal measurements in males **(B)** and 16.35% in females **(D)**.

Implementation of the GFA extracted four latent factors, together explaining 44.5+/-1.0% of variability for males and 55.3+/-0.8% of variability in females in physical-hormone pubertal relationships. Of these four latent factors, two of them explained a large portion of the variability and showed robust components through GFA iterations. The latent factor loadings for the two most robust factors in males and in females are presented in **Figure 5**.

In males, latent factor 1 (LF 1) accounted for 22.87% of the variability among the metrics of physical features and hormones. LF 1 was driven by androgen levels (DHEA and testosterone), and to a lesser extent by skin and body hair, followed by height, facial hair, and voice (**Figure 5A**). Specifically, higher LF 1 scores indicated higher hormone levels with more maturation among physical features, and lower LF 1 scores indicated lower hormone levels with less maturation of physical features. Also in males, latent factor 2 (LF 2; **Figure 5B**) accounted for 15.77% of the variability among the metrics of physical features and hormones, with the strongest loadings observed for body hair, followed by facial hair, skin, voice, and height, with weak loadings for androgen levels. Therefore, LF 2 in males was primarily driven by physical features. Male components 3–4 did not include hormone levels, but rather explained less than 5% of the variability among physical features (4.0 and 1.8%, respectively; data not shown).

For females, highly similar latent factors were extracted compared to males. Latent factor 1 (LF 1; **Figure 5C**) in females explained 31.16% of the variability among physical features and hormone measures. Higher LF 1 scores indicated higher hormone levels with more maturation of body hair, skin, and breast development, and to a lesser extent for menarche and height; indicating LF 1 primarily driven by hormones, as observed in males. Again, similar to males, latent factor 2 (LF 2) for females (**Figure 5D** **)** accounted for 16.35% of the variability among the metrics of physical features and hormones, with the strongest loadings for body hair, skin, breast development, and height as compared to menarche and for estradiol, with DHEA and testosterone demonstrating smaller effects. Together, LF 2 is primarily driven by physical features in females, as was also observed in males. Female components 3–4 did not include hormone levels and only explained 5.0 and 2.9% of variability among physical features, respectively (data not shown). In both males and females, LF 1 and LF 2 were found to be robust, had stable loadings, and were replicated in two split-half sub-samples as well as in follow-up analyses that included BMIz, socioeconomic status, and race/ethnicity as additional grouped variables in GFA iterations (**Supplemental Tables 8–11**).

**Figure 6:**
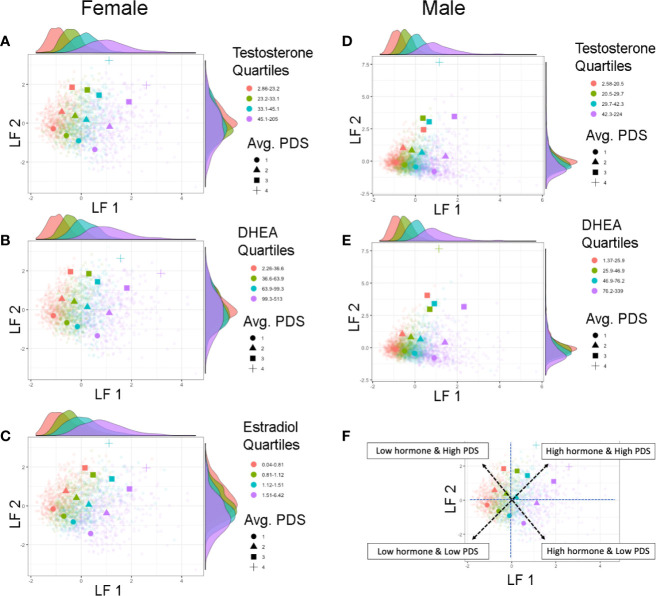
Individual differences in pubertal maturation as characterized by latent factors. Plots show individual scores (lighter, smaller colored shapes) as well as group-means (darker, larger colored shapes) of latent factor 1 (LF 1) and latent factor 2 (LF 2) in females **(A–C)** and males **(D, E)** by average score of physical features reported on the Pubertal Development Scale (PDS) (shape) as well as quartile range (color) of testosterone **(A, D)**, DHEA **(B, E)**, or estradiol **(C)**. Opposite of each axis shows the marginal density plot of each latent factor as a function of each quartile of the given hormone (top: LF 1 by hormone density plot, right: LF 2 by hormone density plot). The correspondence between the two latent factors together captures synchrony and asynchrony between hormones and concomitant perceived physical features across this large child sample **(F)**. Synchronous patterns are represented among individuals with a lower LF 1 and lower LF 2 scores who are pre-pubertal with low hormone levels (pink circle), and among individuals with a higher LF 1 and higher LF 2 scores who are the most advanced in physical maturation with high hormone levels (purple square or cross-hair). Opposing scores between LF 1 and LF 2 (e.g. higher LF 1 but lower LF 2 scores, or lower LF 1 but higher LF 2 scores) indicate a more asynchronous pattern between hormone levels and physical features.

In both sexes, LF 1 scores indicate pubertal maturation as indexed by hormone levels and to a lesser extent concomitant maturation in physical features. LF 2 is orthogonal to LF 1 and is mainly driven by residual increases in maturation of physical features that are not necessarily associated with concomitant increases in pubertal hormones. Interestingly, when evaluating the correspondence between these two latent factors, how synchronous an individual’s pubertal hormone levels are with perceived changes in physical features can be evaluated. Plotting the correspondence between LF 1 and LF 2 scores with each individual’s hormone and average PDS values, unveiled unique patterns reflecting individual differences as captured by these latent factors of pubertal maturation (**Figure 6**). Specifically, the correspondence between LF 1 and LF 2 described a profile of synchronies (e.g., higher scores on both factors, or lower scores on both factors) and asynchronies (a higher score on one factor with a lower score on the other factor) in the pattern of how physical and hormone measures relate to each other. Individuals showing lower LF 1 and LF 2 scores are pre-pubertal based on perceived sexual maturation and also have sex hormones within the bottom 25^th^ percentile of their same-sex peers at 9–10 years of age. In contrast, individuals with higher LF 1 and LF 2 scores are most advanced as evidenced by higher average PDS scores as well as hormone levels within the top 75^th^ percentile of their same-sex peers at 9–10 years of age. However, widespread variability in both LF 1 and LF 2 scores highlight that various patterns of perceived physical features and hormone maturation are rather common, such as having very high hormone levels but perceived physical features that are deemed pre-pubertal (i.e. as indexed by a higher LF 1, but a lower LF 2 score), or even having rather low hormone levels as compared to peers, but more advanced perceived physical features as captured by caregiver report (i.e. as indexed by a lower LF 1, but a higher LF 2 score).

**Figure 7:**
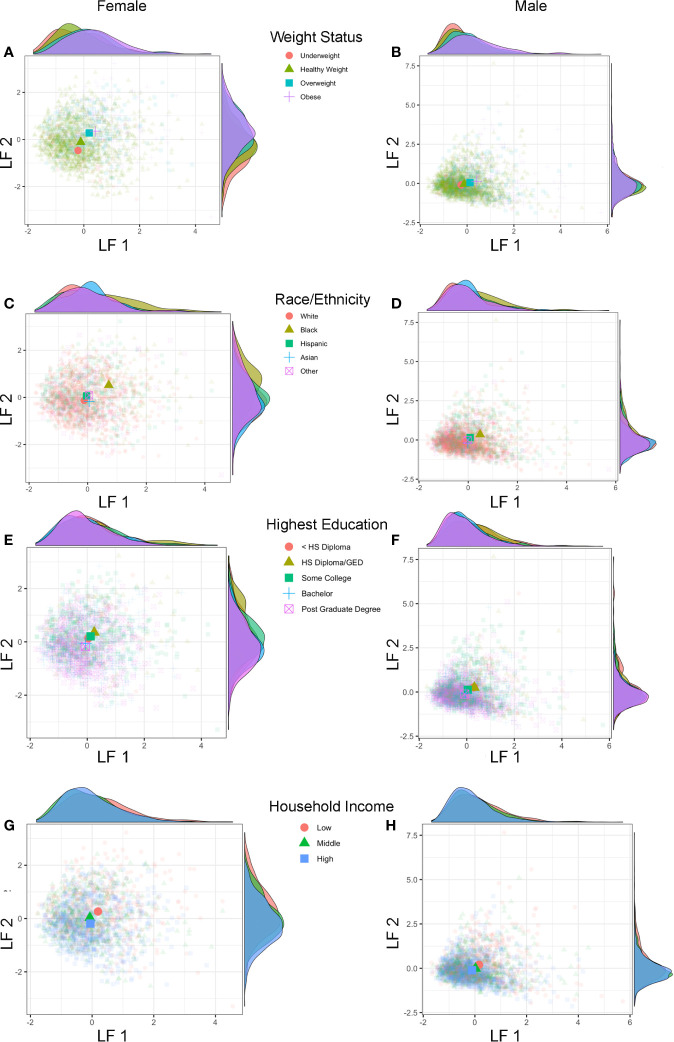
Latent factors and sociodemographic characteristics. Plots show individual scores (lighter, smaller colored shapes) as well as group-means (darker, larger colored shapes) of latent factor 1 (LF 1) and latent factor 2 (LF 2) in females and males by weight status **(A, B)**, race/ethnicity **(C, D)**, highest parental education **(E, F)**, and household income **(G, H)**. Opposite of each axis shows the marginal density plot of each latent factor as a function of each sociodemographic measure. Group differences in pubertal maturation is apparent after integrating hormone levels and physical features using individual latent factors, with more advanced pubertal maturation seen (higher LF 1 and LF 2 scores) for overweight/obese versus underweight/healthy weight **(A, B)** as well as Black versus White, Hispanic, Asian, and Other (multi-race) **(C, D)** with larger effects seen in females as compared to males.

Lastly, we also explored if previously identified sociodemographic differences would also be apparent using the combined estimates of shared variance between physical and hormonal metrics of puberty (LF 1 and LF 2) (**Figure 7**). Group differences were apparent for weight status and race/ethnicity when plotting each subject’s LF 1 and LF 2 (**Figures 7A–D**), but less so for highest education or household income (**Figures 7E–H**). Overweight and obese individuals (as compared to healthy or underweight youth) as well as Black youth (compared to other race/ethnic categories) had overall higher positive LF 1 and LF 2 scores, suggesting greater pubertal maturation as compared to their peers. In summary, unique patterns emerge when examining individual variability in the correspondence between perceived physical features and sex hormone levels in 9–10 year-olds, and sociodemographic differences can also be captured *via* the integration of perceived physical and hormone markers of pubertal maturation *via* GFA.

## Discussion

The present findings contribute to the scientific investigation of pubertal maturation in several meaningful ways, including 1) confirmed sociodemographic differences in pubertal status in a recently collected large sample of 9–10 year-old children in the United States, and 2) the identification of latent variables to integrate physical and hormonal features towards a better understanding of individual differences in pubertal maturation alongside age- and sex- matched peers. Additionally, the present study demonstrates the methodological feasibility of assessing hormone levels on this historically large scale in youth, and during the onset of pubertal maturation when levels are just beginning to rise, using a non-invasive biological specimen: saliva.

Confirmation of sociodemographic differences in pubertal status is important in the ABCD sample and in the narrow-age range for the following reasons: 1) ABCD is a publicly available data set, with an unprecedented release time, making utilization of this sample for investigation of pubertal variables very feasible for independent research groups across the world; and, 2) it confirms previous research findings for sociodemographic associations with pubertal maturation in a large, diverse sample of today’s children in the United States. As is widely accepted in the literature (28, 57), on average, females were of a more advanced pubertal stage at 9–10 years as compared to males, across a wide range of pubertal measures (e.g., overall perceived physical features and adrenal- and gonadal-related physical features), with larger non-linear increases in androgen levels seen with age in females as compared to males. As previously found, pubertal maturation was more advanced among Black youth (18, 19), and related to weight status (20–22) and (to a lesser degree in the present study) factors reflecting socioeconomic status (16, 55). Interestingly, these associations were more pronounced in females. The larger associations between sociodemographic measures and pubertal maturation in females as compared to males could possibly be due to the increased variability in pubertal measures among females compared to males within this narrow age range and should be investigated in future ABCD releases as the children get older. Alternatively, it may also be plausible that biological mechanisms underlying pubertal maturation may be differentially affected by sociodemographic characteristics in males versus females (55), which also warrants future investigation.

The accuracy of measurement *via* self or caregiver-report of physical maturation can vary based on the child’s sex, weight status, and race/ethnicity (58, 59); thus, salivary hormone levels in the current study are a complementary objective measurement. In fact, salivary hormone levels, including DHEA, testosterone, and estradiol, largely confirm sociodemographic differences as seen by caregiver report on the PDS. As such, our results indicate that sociodemographic measures of race/ethnicity and socioeconomic status are associated with pubertal measures in a diverse sample of youth and that this relationship is not entirely due to differences in BMI. Given that the existing literature has found that normal onset of pubertal maturation tends to occur 1–2 years earlier in females as compared to males (60), it was unsurprising that approximately 2/3 of females but only 1/3 of males were at the early pubertal stage or later. Given the less overt visible signs of early stages of puberty in boys (2), the higher levels of DHEA in females compared to males in the current study provide harmonizing evidence of sex differences in pubertal onset.

The current findings also show that while accounting for each variable during statistical modeling, race/ethnicity and body weight status both uniquely related to more advanced physical and hormonal makers of maturation in the ABCD cohort. As such, our results are aligned with previous findings that Black youth develop secondary sex characteristics earlier as compared to White youth (24). Also, the racial and ethnic differences we found are congruent with previous reports from large datasets [e.g., PROS (24, 46), and NHANES (61) datasets]. For example, in PROS’s report, approximately 68% of White and 95% of Black females were found to display breast and/or pubic hair development by age 10 as determined by a physician. Prevalence of pubic hair in PROS was 26% for White, 54% for Black, and 22% for Hispanic males by age 10 (24). Previous studies did not include Asian or Multi-race/Other as individual groups, yet we found a similar prevalence of 26.7 and 28.6% for males and 62.4 and 72.4% for females having started the pubertal process for Asian and Multi-race/Other groups, respectively.

We found the highest averaged levels of DHEA, testosterone, and estradiol (in females) in Black youth as compared to all other racial and ethnic groups. It is still unclear why broad racial groupings consistently relate to pubertal metrics. Importantly, we must consider that the broad categories for race and ethnicity in this study are neither biological nor genetic factors, but also serve as proxies for a number of co-occurring factors, including environmental factors, which together may influence the biological etiology of pubertal maturation. Significant results pertaining to these broad race and ethnicity groupings in this study may also pertain to selection bias and omitted variables underlying the study sampling technique. Elucidating the relationship between pubertal maturation and co-occurring factors that may be confounded with race and ethnicity is a grossly understudied area that requires extensive and exclusive attention in future studies designed to answer this line of inquiry.

In addition, the current study lends strong support for the need to consider socioeconomic status and obesity in studying pubertal development; especially given that each accounted for unique variance in physical and hormonal markers of maturation in the ABCD cohort. Although findings have suggested overweight or obese status may lead to early initiation of pubertal development in females (62), fewer studies have been conducted in males and with mixed findings regarding an earlier onset versus delay in the initiation of the pubertal process (21). More advanced physical maturation, including higher androgen levels, were seen in overweight and obese children of both sexes in the ABCD cohort, albeit effects were larger for females. These findings corroborate previous findings of increased testosterone levels in obese prepubertal and pubertal girls from Germany (63) and in the United States (62), as well as higher DHEA levels in obese prepubertal boys in China as compared to normal weight peers (23). Less mature patterns of physical maturation in relation to higher socioeconomic position have also been noted before in other cohorts (55). Future studies using the ABCD cohort are warranted to determine if these associations may be due to other family or neighborhood-wide social and environmental factors (64), stress, or exposure to endocrine disruptors, which may disproportionately affect low-income families and their communities [or specific races *via* unregulated beauty products containing endocrine disruptors (65)]. Physical and hormonal changes do not occur in isolation during puberty: there is a bi-directional cross-talk between the Hypothalamic Pituitary Gonadal (HPG)-axis and the Hypothalamic Pituitary Adrenal (HPA) stress axis in later stages of pubertal maturation, with each system thought to be sensitive to physical and social stressors (66). As the ABCD Study progresses, the breadth of data as well as the longitudinal design will provide the opportunity to examine how sociodemographic and environmental factors map onto temporal patterns of pubertal maturation across adolescence; ultimately strengthening our understanding of the potential causal relationships between environmental factors and pubertal timing and tempo (67).

The similarity of patterns seen in how physical and hormone markers of puberty relate to sociodemographic characteristics highlights agreement in physical and hormone markers at early stages of pubertal maturation. However, we derived latent factors to further characterize the individual variability in pubertal maturation and capture the multifaceted, physical, and hormonal cascade of the early stages of the pubertal process. Two puberty related latent factors emerged for both males and females, the first driven by hormone levels and the second driven by perceived physical features. The correspondence between the two factors described a profile of synchronies (e.g., a higher score on both factors, or a negative score on both factors) and asynchronies (e.g., a higher score on one factor with a lower score on the other factor) in the relationship between physical features and hormone levels. Importantly, these two factors showed stability under multiple iterations accounting for potential confounds of sociodemographic measures. Further, while neither puberty related factor was driven by sociodemographic differences, factor scores were associated with sociodemographic measures such that more advanced patterns of pubertal maturation were observed in youth identifying as Black, as well as youth with overweight or obese status regardless of race. Future studies are needed to understand the mechanisms by which individual variability and patterns of synchrony emerge in pubertal maturation and whether these patterns remain stable or change with time. Possible mechanisms include variability in tissue sensitivity to hormones (68) such that some individuals may exhibit a higher degree of physical maturation with lower levels of hormones, genetic factors, or variability in environmental factors contributing to relative differences in hormones and/or physical features of maturation (69). Lastly, the hormone-related factor explained the most variability among all pubertal measures and this may speak to possible differences in the sensitivity of each metric to assess puberty during its early stages, with potential greater sensitivity at the level of hormones versus the less sensitive PDS measure *via* self or caregiver-report.

From a methodological perspective, the present findings validate salivary sampling on a large scale, as age- and sex-associated patterns in hormone levels were congruent with theoretical predictions for a population of 9–10 year-olds who primarily have not yet begun or are in early stages of pubertal maturation. This is the largest salivary investigation of early puberty in adolescents to date, thus demonstrating the feasibility of collecting meaningful saliva-based hormone levels on this large scale as a significant contribution to the field of salivary biosciences. Salivary sampling is less invasive than blood and can be collected in a variety of settings by trained research staff or even self-sampled outside of the laboratory allowing for increased ecological validity of salivary measures in the future. Methodological measures that were expected to influence hormone values were observed in the present study, including: a negative association between time of collection and testosterone levels, and positive associations between estradiol in girls with time of collection, collection duration, and caffeine consumption. These methodological considerations (e.g., time of day or time since waking) are predicted to exert larger effects on hormone levels with increased maturation of the HPG axis in later stages of puberty, at which time adult-like circadian patterns become more established (70). As hormones rise rapidly in future assessments in the ABCD cohort, longitudinal analyses controlling for these methodological factors around saliva collection will become increasingly important and likely explain some variance in absolute hormone levels unrelated to pubertal maturation. For example, potential changes in collection times within and between participants across years may contribute to artificial declines/increases in hormone levels due to circadian fluctuations. Here, salivary hormone levels are not being proposed for diagnostic purposes, but rather as guides for expected ranges of hormone values among 9–10 year olds participating in research studies. In fact, this large-scale study provides more validation for salivary hormone levels as important tools for studying child development within the realm of research. In past research, salivary estradiol levels have received less attention than testosterone or DHEA, and have historically been considered too low in pre or early pubertal children to be utilized as a reliable tool for clinical diagnosis, regardless of sampling blood or saliva (71). To the contrary, we believe what is largely missing from the literature is a large enough data set for salivary hormones in typically developing children to serve as “reference ranges or norms”, particularly for estradiol.

Limitations of the current analyses should be noted. The ABCD Study implemented the PDS measurement of pubertal maturation given its advantage of being brief and relatively noninvasive and validated compared to traditional Tanner Staging by physician, and that the PDS it is widely used in the literature. However, PDS values can be biased based on self-report by caregiver or child (72, 73). The ABCD Study does not capture the transition of individuals into puberty for some of the earliest developers, particularly females who identify as Black. In addition, testicular volume is a hallmark of early HPG activation and puberty onset in males. Thus, PDS may not adequately capture earlier pubertal events in boys (1). Therefore, a physical exam by a qualified physician is required for an objective assessment of the earliest stages of puberty in both sexes.

In ABCD, we relied on self- and caregiver- report that reflect “perceived” physical pubertal development rather than relying on clinician ratings which would have been too cumbersome and costly in such a large sample in light of all the other time consuming evaluations. Using this measurement, the majority of the females in the sample (96%) had yet to complete the key pubertal milestone of menarche at baseline; however, approximately 10 females were reported to have experienced menarche rather early at ages 7–8 years. The current study did not collect information regarding potential evaluation by a pediatric endocrinologist to determine if these individuals would meet clinical criteria for extreme cases of early puberty, including precocious puberty (as defined as occurring in females <8 years or males <9 years) that affects as many as 1:5,000 children (74). It is also important to note that caregivers and youth may overestimate perceived growth using the PDS. For example, caregivers considered 51% of males and 63% of females to have a growth spurt that was underway. Peak height velocity typically occurs between ages 10–14 years for females and age 12–16 years for males (13). Thus, future analyses using the ABCD dataset may benefit from calculation of peak height velocity using objective anthropomorphic measures as well as examination of age at menarche in females, as these metrics may be a more objective measure of pubertal timing (75).

A primary limitation of this study is the cross-sectional nature of the data utilized, particularly given the longitudinal and dynamic nature of pubertal maturation. In an Australian cohort where hormone levels were assayed from urine across 3 years from age 11–14 years, two phenotypes of longitudinal hormone trajectories were found: either “smoother” or “bumpy” (76). Future analyses using ABCD salivary pubertal hormone markers longitudinally as they become available will help interpret similarities or differences in observed trajectories measured in urine or blood.

Regarding limitations of salivary hormone levels in this study, the oral environment can impact accuracy of measurement of analytes, resulting in an over- or under-estimation of unbound steroid hormone levels relative to bound levels. Despite these caveats, correlations between blood levels with saliva using immunoassays to assess DHEA, testosterone, and estradiol can vary across time of day, pubertal stage, and context and are in general 0.86, 0.96, and 0.80, respectively (28, 30, 77–81). Correlations between serum and saliva reflect differences in methodology, sensitivity of specimen, and assessment methodology, as well as mechanistically relating to how hormones arrive in the blood versus saliva. Blood is often considered the clinical gold standard for accuracy of biomarker levels, whereas saliva has unique benefits that make it feasible to be utilized in any ecological context, including self-assessment in the absence of a phlebotomist. Estradiol with immunoassay may have reduced sensitivity to pubertal maturation during the earliest stages, making associations of estradiol and physical maturation less meaningful early on (as in the present analyses) and more meaningful with future longitudinal time points in ABCD. DHEA sulfate (DHEA-S) is a key hormone precursor to DHEA, then testosterone, then estradiol, and was not assayed in collected saliva in ABCD. Future studies that include DHEA-S (potentially within the ABCD cohort) may provide a more comprehensive panel to determine changes in hormone metabolism across pubertal development.

In conclusion, the present findings characterize associations between physical and hormonal sexual maturation in 9–10 year-old children and integrate key sociodemographic information in mapping early stages of sexual maturation. Sociodemographic differences in physical and hormonal markers highlight the complex interplay between racial and ethnicity backgrounds, physical health, and socioeconomic factors at the early stages of pubertal maturation. Further, this study demonstrates feasibility and validity of salivary hormone sampling on a large scale and provides a novel approach in characterizing complex pubertal maturational patterns as seen by various measurements. Application of a factor analysis approach (GFA) to the pubertal research niche allowed us to reduce the dimensionality across several measures for hormones and physical features to produce two factors, which together can help to understand a large degree of the individual variability in how physical features relate to hormone levels at ages 9–10 years, and where an individual’s pattern in this relationship between multiple metrics of puberty is located relative to age- and sex- matched peers. Through future exploration of how physical and hormonal markers relate to each other and in connection to a wide range of developmental outcomes, the ABCD Study holds great potential for rapid advancement in understanding the universal biological phenomenon of puberty and how it impacts both typical and pathological pediatric health trajectories moving forward.

## Data Availability Statement

The datasets presented in this study can be found in online repositories. The names of the repository/repositories and accession number(s) can be found below: Data used in the preparation of this article were obtained from the Adolescent Brain Cognitive Development (ABCD) Study^SM^ (https://abcdstudy.org), held in the NIMH Data Archive (NDA). This is a multisite, longitudinal study designed to recruit more than 10,000 children aged 9–10 and follow them over 10 years into early adulthood. The ABCD Study® is supported by the National Institutes of Health and additional federal partners under award numbers U01DA041022, U01DA041028, U01DA041048, U01DA041089, U01DA041106, U01DA041117, U01DA041120, U01DA041134, U01DA041148, U01DA041156, U01DA041174, U24DA041123, and U24DA041147. A full list of supporters is available at https://abcdstudy.org/federal-partners.html. A listing of participating sites and a complete listing of the study investigators can be found at https://abcdstudy.org/consortium_members/. ABCD consortium investigators designed and implemented the study and/or provided data but did not necessarily participate in analysis or writing of this report. This manuscript reflects the views of the authors and may not reflect the opinions or views of the NIH or ABCD consortium investigators. The ABCD data repository grows and changes over time. The ABCD data used in this report came from 10.15154/1504041.

## Ethics Statement

The current study involving human participants was reviewed and approved by a centralized institutional review board (IRB) at the University of California, San Diego. Study sites obtained approval from their local IRBs. Written informed consent to participate in this study was provided by the participants’ legal guardian/next of kin.

## Author Contribution

MeH, KU, FB, DB, GD, MG, EH, TJ, ES, ST, and SW contributed to the conception of this study. AMa, KU, MG, FB, AA, KB, DB, AB-S, BC, LCo, CC, TC, RD, GD, JD, SE, TE, AG, KG, CH, RH, LH, TJ, NK, AL, KeL, KLi, ML, BL, HM, AMa, MM, BN, GN, CP, MP, AP, KR, NR, GR, KS, PS, MS, RT, and RZ contributed by critically revising the work. AMa, KA, MA, MB, DB, FB, BC, BC, LCh, DC, CC, TC, MD, AD, ND, JD, SE, TE, DF, EF, HG, DGe, PG, AG, KG, SH, AH, MHe, JH. CH, RH, MHu, MI, JJ, TJ, AL, KLi, ML, EM, BN, AP, LP, GR, PS, ES, LS, MS, ST, NW, DY, and RZ contributed to the collection of data. MeH, KU, EK, MG, and WT performed all analyses of the data. MeH, KU, MG, FB, ES, and WT contributed to the interpretation of the work. MeH, KU, and MG drafted the manuscript. All authors contributed to the article and approved the submitted version.

## Funding

Financial support was provided by the National Institutes of Health under award numbers: K01MH108761 to MH and K01AA026889 to KAU. The ABCD Study is supported by the National Institutes of Health and additional federal partners under award numbers U01DA041022, U01DA041028, U01DA041048, U01DA041089, U01DA041106, U01DA041117, U01DA041120, U01DA041134, U01DA041148, U01DA041156, U01DA041174, U01DA041025, U24DA041123, and U24DA041147. The funding sources had no involvement in statistical analysis and interpretation; writing the report; or the decision to submit the article for publication.

## Conflict of Interest

In the interest of full disclosure, DG is founder and chief scientific and strategy advisor at Salimetrics LLC and Salivabio LLC (Carlsbad, CA) and these relationships are managed by the policies of the committee’s on conflict of interest at Johns Hopkins University School of Medicine and the University of California at Irvine. ND and DF have a financial interest in Nous Imaging Inc. and may financially benefit if the company is successful in marketing FIRMM software products. DF is a patent holder on the Framewise Integrated Real-Time Motion Monitoring (FIRMM) software and is a co-founder of Nous Imaging Inc. KG provides consultation to Pfizer, Inc. MP is an advisor to Spring Care, Inc., a behavioral startup and has received royalties for an article about methamphetamine in Uptodate. Authors AG and FB were employed by the company SRI International. SW has stock ownership in GE and Merck.

All other authors declare that the research was conducted in the absence of any commercial or financial relationships that could be construed as a potential conflict of interest.
