# Supplementary material for: Correspondence Between Perceived Pubertal Development and Hormone Levels in 9-10 Year-Olds From the Adolescent Brain Cognitive Development Study
Source: Front Endocrinol (Lausanne). 2021 Feb 18;11:549928. doi: 10.3389/fendo.2020.549928 (PMC7930488; doi:10.3389/fendo.2020.549928)

**Supplementary Material**

**Title: Correspondence Between Perceived Pubertal Development and Hormone Levels in 9-10 Year-Olds From the Adolescent Brain Cognitive Development Study**

**Authors: Megan M. Herting*, Kristina A. Uban*, Marybel Robledo Gonzalez, Fiona C. Baker, Eric C. Kan, et al.**

**Supplementary Table 1.** Pubertal Development Scale summary measures for male and female youth based on caregiver or youth self-report.

**Supplementary Table 2.** Caregiver-youth agreement for Pubertal Development Scale items and summary scores by sex and in relation to the biological mother or biological father as caregiver reporting.

**Supplementary Table 3.** Polychoric pairwise correlations for caregiver report of physical features and summary measurements, as well as hormone levels for males and females.

**Supplementary Table 4.** Post-hoc comparisons for sociodemographic differences in the average Pubertal Development Scale score for males and females based on caregiver report.

**Supplementary Table 5.** Post-hoc comparisons for sociodemographic differences in DHEA levels for males and females.

**Supplementary Table 6.** Post-hoc comparisons for sociodemographic differences in Testosterone levels for males and females.

**Supplementary Table 7.** Post-hoc comparisons for sociodemographic differences in Estradiol levels for females only.

**Supplementary Table 8.** Latent factor 1 median loadings and 95% CI for males.

**Supplementary Table 9.** Latent factor 2 median loadings and 95% CI for males.

**Supplementary Table 10.** Latent factor 1 median loadings and 95% CI for females.

**Supplementary Table 11.** Latent factor 2 median loadings and 95% CI for females.

**Supplementary Figure 1.** DHEA and age associations in females (F) and males (M).

**Supplementary Figure 2.** Testosterone and age associations in females (F) and males (M).

**Supplementary Figure 3.** Estradiol and age associations in females (F) only.

**Supplementary Table 1.** Pubertal Development Scale summary measures for male and female youth based on caregiver or youth self-report. A minimum number of questions were required for a valid calculation of each metric. Number of subject's not meeting the minimum number of question are listed as non-valid. The number of subjects with valid estimates are presented along with the mean and (standard deviation, sd) or frequency (%) of each category.

|  | **Caregiver Report** | | | | | | **Youth Report** | | | | | |
| --- | --- | --- | --- | --- | --- | --- | --- | --- | --- | --- | --- | --- |
|  | **Male** | | | **Female** | | | **Male** | | | **Female** | | |
|  | Non-valid N | Valid N | Mean (sd) | Non-valid N | Valid N | Mean (sd) | Non-valid N | N | Mean (sd) | Non-valid N | N | Mean (sd) |
| Average PDS | 90 | 6051 | 1.4 (0.38) | 97 | 5562 | 1.8 (0.53) | 880 | 5278 | 1.7 (0.47) | 1321 | 4307 | 1.7 (0.51) |
| Gondal Score | 255 | 5873 | 1.5 (0.36) | 286 | 5365 | 1.8 (0.49) | 2275 | 3822 | 1.7 (0.49) | 2866 | 2635 | 1.8 (0.53) |
| Adrenal Score | 199 | 5908 | 1.4 (0.56) | 169 | 5457 | 1.7 (0.73) | 1097 | 5061 | 1.6 (0.63) | 973 | 4655 | 1.7 (0.66) |
| PDS Category | 206 | 5935 |  | 199 | 5460 |  | 694 | 5464 |  | 1560 | 4068 |  |
| Pre |  |  | 70.06% |  |  | 30.74% |  |  | 29.94% |  |  | 25.57% |
| Early |  |  | 24.06% |  |  | 23.61% |  |  | 47.62% |  |  | 26.84% |
| Mid |  |  | 5.31% |  |  | 43.00% |  |  | 20.46% |  |  | 44.22% |
| Late |  |  | 0.49% |  |  | 2.55% |  |  | 1.78% |  |  | 3.10% |
| Post |  |  | 0.07% |  |  | 0.11% |  |  | 0.20% |  |  | 0.27% |

**Supplementary Table 2.**

Caregiver-youth agreement for Pubertal Development Scale items and summary scores by sex and in relation to the biological mother or biological father as caregiver reporting. Kappa coefficient means (95% confidence intervals, CI) as well as polychoric coefficients rho (standard error, SE).

| **Males** | | | | | | |
| --- | --- | --- | --- | --- | --- | --- |
|  | Biological Mothers | | | Biological Fathers | | |
| **PDS Items** | N | Kappa (95% CI) | Rho (SE) | N | Kappa (95% CI) | Rho (SE) |
| Height | 3291 | 0.15 (0.12, 0.18) | 0.17 (0.02) | 403 | 0.24 (0.15, 0.34) | 0.28 (0.06) |
| Body Hair | 4804 | 0.13 (0.10, 15) | 0.21 (0.02) | 592 | 0.16 (0.8, 0.24) | 0.24 (0.06) |
| Skin Changes | 4334 | 0.19 (0.16, 0.22) | 0.31 (0.02) | 536 | 0.23 (0.14, 0.33) | 0.37 (0.06) |
| Voice Change | 4853 | 0.10 (0.07, 0.12) | 0.30 (0.03) | 600 | 0.09 (0.03, 0.16) | 0.24 (0.08) |
| Facial Hair | 4879 | 0.15 (0.11, 0.18) | 0.39 (0.03) | 593 | 0.12 (0.2, 0.22) | 0.33 (0.09) |
| **PDS Summary Scores** | N | Kappa (95% CI) | Rho (SE) | N | Kappa (95% CI) | Rho (SE) |
| Average PDS | 4390 | 0.19 (0.16, 0.22) | 0.22 (0.02) | 524 | 0.25 (0.16, 0.33) | 0.31 (0.04) |
| Gondal Score | 3081 | 0.17 (0.14, 0.20) | 0.22 (0.02) | 378 | 0.25 (0.15, 0.34) | 0.31 (0.05) |
| Adrenal Score | 4101 | 0.17 (0.14, 0.20) | 0.25 (0.02) | 504 | 0.23 (0.14, 0.32) | 0.32 (0.05) |
| PDS Category | 4450 | 0.05 (0.03, 0.8) | 0.25 (0.02) | 546 | 0.05 (-0.02, 0.11) | 0.24 (0.06) |
| **Females** | | | | | | |
|  | Biological Mothers | | | Biological Fathers | | |
| **PDS Items** | N | Kappa (95% CI) | Rho (SE) | N | Kappa (95% CI) | Rho (SE) |
| Height | 2632 | 0.22 (0.19, 0.26) | 0.28 (0.02) | 323 | 0.25 (0.15, 0.35) | 0.30 (0.06) |
| Body Hair | 4333 | 0.38 (0.35, 0.40) | 0.47 (0.02) | 527 | 0.37 (0.29, 0.46) | 0.47 (0.04) |
| Skin Changes | 3839 | 0.53 (0.34, 0.40) | 0.46 (0.02) | 503 | 0.37 (0.28, 0.45) | 0.47 (0.05) |
| Breast Development | 3839 | 0.53 (0.50, 0.55) | 0.63 (0.01) | 478 | 0.50 (0.43, 0.57) | 0.60 (0.04) |
| Menarche | 4211 | 0.80 (0.74, 0.85) | 0.98 (0.01) | 517 | 0.86 (0.73, 1.0) | 0.99 (0.01) |
| **PDS Summary Scores** | N | Kappa (95% CI) | Rho (SE) | N | Kappa (95% CI) | Rho (SE) |
| Average PDS | 3569 | 0.53 (0.50, 0.55) | 0.52 (0.01) | 436 | 0.54 (0.46, 0.63) | 0.54 (0.04) |
| Gondal Score | 2112 | 0.55 (0.51, 0.59) | 0.55 (0.02) | 259 | 0.56 (0.45, 0.66) | 0.56 (0.05) |
| Adrenal Score | 3791 | 0.44 (0.41, 0.47) | 0.49 (0.01) | 467 | 0.44 (0.45, 0.66) | 0.51 (0.04) |
| PDS Category | 3319 | 0.23 (0.19, 0.26) | 0.67 (0.01) | 411 | 0.20 (0.09, 0.31) | 0.68 (0.04) |

**Supplementary Table 3.**

Polychoric pairwise correlations for caregiver report of physical features and summary measurements, as well as hormone levels for males and females. NA denotes failed correlation due to limited variance for the pubertal category variable in males.

|  | Height | Body Hair | Skin | Voice | Facial Hair | Average PDS | Gonadal Score | | Adrenal Score | | Pubertal Category | | DHEA | | Testosterone | |  | | |
| --- | --- | --- | --- | --- | --- | --- | --- | --- | --- | --- | --- | --- | --- | --- | --- | --- | --- | --- | --- |
| Height | -- | -- | -- | -- | -- | -- | -- | | -- | | -- | | -- | | -- | |  | | |
| Body Hair | 0.31 | -- | -- | -- | -- | -- | -- | | -- | | -- | | -- | | -- | |  | | |
| Skin | 0.20 | 0.39 | -- | -- | -- | -- | -- | | -- | | -- | | -- | | -- | |  | | |
| Voice | 0.14 | 0.26 | 0.24 | -- | -- | -- | -- | | -- | | -- | | -- | | -- | |  | | |
| Facial Hair | 0.10 | 0.34 | 0.27 | 0.33 | -- | -- | -- | | -- | | -- | | -- | | -- | |  | | |
| Average PDS | 0.69 | 0.75 | 0.67 | 0.49 | 0.49 | -- | -- | | -- | | -- | | -- | | -- | |  | | |
| Gonadal Score | 0.87 | 0.42 | 0.30 | 0.53 | 0.47 | 0.85 | -- | | -- | | -- | | -- | | -- | |  | | |
| Adrenal Score | 0.30 | 0.85 | 0.81 | 0.30 | 0.37 | 0.85 | 0.44 | | -- | | -- | | -- | | -- | |  | | |
| Pubertal Category | 0.40 | *NA* | 0.40 | 0.46 | 0.45 | *NA* | 0.67 | | *NA* | | -- | | -- | | -- | |  | | |
| DHEA | 0.11 | 0.15 | 0.17 | 0.06 | 0.06 | 0.19 | 0.13 | | 0.19 | | 0.18 | | -- | | -- | |  | | |
| Testosterone | 0.10 | 0.14 | 0.15 | 0.07 | 0.09 | 0.18 | 0.14 | | 0.18 | | 0.18 | | 0.71 | | -- | |  | | |
| **Females** | | | | | | | | | | | | | | | | | |  |  |
|  | Height | Body Hair | Skin | Breast | Menarche | Average PDS | Gonadal Score | Adrenal Score | | Pubertal Category | | DHEA | | Testosterone | | Estradiol | | |  |
| Height | -- | -- | -- | -- | -- | -- | -- | -- | | -- | | -- | | -- | | -- | | |  |
| Body Hair | 0.33 | -- | -- | -- | -- | -- | -- | -- | | -- | | -- | | -- | | -- | | |  |
| Skin | 0.27 | 0.52 | -- | -- | -- | -- | -- | -- | | -- | | -- | | -- | | -- | | |  |
| Breast | 0.33 | 0.52 | 0.47 | -- | -- | -- | -- | -- | | -- | | -- | | -- | | -- | | |  |
| Menarche | 0.09 | 0.22 | 0.18 | 0.22 | -- | -- | -- | -- | | -- | | -- | | -- | | -- | | |  |
| Average PDS | 0.61 | 0.80 | 0.74 | 0.77 | 0.42 | -- | -- | -- | | -- | | -- | | -- | | -- | | |  |
| Gonadal Score | 0.74 | 0.54 | 0.46 | 0.80 | 0.50 | 0.89 | -- | -- | | -- | | -- | | -- | | -- | | |  |
| Adrenal Score | 0.34 | 0.89 | 0.85 | 0.57 | 0.23 | 0.89 | 0.57 | -- | | -- | | -- | | -- | | -- | | |  |
| Pubertal Category | 0.40 | 0.82 | 0.54 | 0.85 | 0.46 | 0.92 | 0.83 | 0.80 | | -- | | -- | | -- | | -- | | |  |
| DHEA | 0.11 | 0.29 | 0.29 | 0.28 | 0.15 | 0.34 | 0.26 | 0.34 | | 0.33 | | -- | | -- | | -- | | |  |
| Testosterone | 0.14 | 0.30 | 0.27 | 0.27 | 0.14 | 0.34 | 0.26 | 0.33 | | 0.32 | | 0.75 | | -- | | -- | | |  |
| Estradiol | 0.01 | 0.11 | 0.11 | 0.12 | 0.08 | 0.13 | 0.10 | 0.13 | | 0.13 | | 0.51 | | 0.55 | | -- | | |  |

**Supplementary Table 4.**

Post-hoc comparisons for sociodemographic differences in the average Pubertal Development Scale score for males and females based on caregiver report.

| **Weight Status Post-hoc Contrasts** | **Estimate** | **SE** | **df** | **T** | **p value** |
| --- | --- | --- | --- | --- | --- |
| Male, Underweight - Male, Overweight | -0.100907933 | 0.030913646 | 1632 | -3.264187346 | **0.025** |
| Male, Underweight - Male, Obese | -0.144955375 | 0.030924431 | 1632 | -4.68740632 | **<0.001** |
| Male, Overweight - Male, Obese | -0.044047441 | 0.018625723 | 1632 | -2.364871484 | 0.260 |
| Male, Healthy Weight - Male, Underweight | 0.03839106 | 0.028452801 | 1632 | 1.349289301 | 0.880 |
| Male, Healthy Weight - Male, Overweight | -0.062516874 | 0.015021233 | 1632 | -4.161900205 | **0.001** |
| Male, Healthy Weight - Male, Obese | -0.106564315 | 0.01491431 | 1632 | -7.145105385 | **<0.001** |
| Female, Underweight - Female, Overweight | -0.468731541 | 0.030133844 | 1632 | -15.55498681 | **<0.001** |
| Female, Underweight - Female, Obese | -0.492419779 | 0.030450784 | 1632 | -16.17100471 | **<0.001** |
| Female, Overweight - Female, Obese | -0.023688239 | 0.01970974 | 1632 | -1.201854445 | 0.932 |
| Female, Healthy Weight - Female, Underweight | 0.221999612 | 0.027219911 | 1632 | 8.155780337 | **<0.001** |
| Female, Healthy Weight - Female, Overweight | -0.246731928 | 0.015744781 | 1632 | -15.67071182 | **<0.001** |
| Female, Healthy Weight - Female, Obese | -0.270420167 | 0.016216972 | 1632 | -16.67513304 | **<0.001** |
| **Race/Ethnicity Post-hoc Contrasts** | **Estimate** | **SE** | **df** | **T** | **p value** |
| Male, White - Male, Other | -0.027947853 | 0.018822765 | 1632 | -1.484789938 | 0.898 |
| Male, White - Male, Black | -0.218012329 | 0.019172922 | 1632 | -11.37084546 | **<0.001** |
| Male, White - Male, Asian | 0.012799551 | 0.03997627 | 1632 | 0.320178727 | 1.000 |
| Male, Hispanic - Male, White | 0.060159499 | 0.016806607 | 1632 | 3.579514915 | **0.013** |
| Male, Hispanic - Male, Other | 0.032211646 | 0.021964627 | 1632 | 1.466523729 | 0.905 |
| Male, Hispanic - Male, Black | -0.15785283 | 0.02079544 | 1632 | -7.590742558 | **<0.001** |
| Male, Hispanic - Male, Asian | 0.07295905 | 0.041902407 | 1632 | 1.741166113 | 0.772 |
| Male, Black - Male, Other | 0.190064476 | 0.023642992 | 1632 | 8.038935093 | **<0.001** |
| Male, Black - Male, Asian | 0.23081188 | 0.043148771 | 1632 | 5.349211 | **<0.001** |
| Male, Asian - Male, Other | -0.040747404 | 0.042811402 | 1632 | -0.951788599 | 0.995 |
| Female, White - Female, Other | -0.102016314 | 0.019332202 | 1632 | -5.277014713 | **<0.001** |
| Female, White - Female, Black | -0.318317847 | 0.019576776 | 1632 | -16.25997312 | **<0.001** |
| Female, White - Female, Asian | -0.024961875 | 0.040137938 | 1632 | -0.621902282 | 1.000 |
| Female, Hispanic - Female, White | 0.023423735 | 0.017465102 | 1632 | 1.341173686 | 0.944 |
| Female, Hispanic - Female, Other | -0.07859258 | 0.022460712 | 1632 | -3.499113539 | **0.017** |
| Female, Hispanic - Female, Black | -0.294894112 | 0.021064555 | 1632 | -13.99954136 | **<0.001** |
| Female, Hispanic - Female, Asian | -0.001538141 | 0.041992356 | 1632 | -0.036629061 | 1.000 |
| Female, Black - Female, Other | 0.216301533 | 0.023930141 | 1632 | 9.038874088 | **<0.001** |
| Female, Black - Female, Asian | 0.293355972 | 0.04333539 | 1632 | 6.769431923 | **<0.001** |
| Female, Asian - Female, Other | -0.077054439 | 0.042924684 | 1632 | -1.795107886 | 0.738 |
| **Highest Education Post-hoc Contrasts** | **Estimate** | **SE** | **df** | **T** | **p value** |
| Male, Some College - Male, Graduate Degree | 0.065296104 | 0.016810613 | 1632 | 3.884219048 | **0.004** |
| Male, Some College - Male, Bachelor | 0.048537009 | 0.016494131 | 1632 | 2.942683545 | 0.095 |
| Male, HS Diploma/GED - Male, Some College | 0.03365868 | 0.022165535 | 1632 | 1.518514269 | 0.885 |
| Male, HS Diploma/GED - Male, Graduate Degree | 0.098954785 | 0.02464132 | 1632 | 4.015807019 | **0.002** |
| Male, HS Diploma/GED - Male, Bachelor | 0.082195689 | 0.024250692 | 1632 | 3.389416181 | **0.025** |
| Male, HS Diploma/GED - Male, <HS Diploma | 0.013434388 | 0.034224842 | 8831 | 0.392533233 | 1.000 |
| Male, Bachelor - Male, Graduate Degree | 0.016759095 | 0.014404365 | 1632 | 1.163473342 | 0.978 |
| Male, <HS Diploma - Male, Some College | 0.020224293 | 0.031351468 | 1632 | 0.645082783 | 1.000 |
| Male, <HS Diploma - Male, Graduate Degree | 0.085520397 | 0.033580725 | 1632 | 2.546710876 | 0.244 |
| Male, <HS Diploma - Male, Bachelor | 0.068761301 | 0.033232863 | 1632 | 2.069075475 | 0.550 |
| Female, Some College - Female, Graduate Degree | 0.080213792 | 0.01810965 | 1632 | 4.429339597 | **<0.001** |
| Female, Some College - Female, Bachelor | 0.065117907 | 0.017602326 | 1632 | 3.699392141 | **0.008** |
| Female, HS Diploma/GED - Female, Some College | 0.001985359 | 0.023165708 | 1632 | 0.085702516 | 1.000 |
| Female, HS Diploma/GED - Female, Graduate Degree | 0.082199151 | 0.026103806 | 1632 | 3.148933525 | 0.053 |
| Female, HS Diploma/GED - Female, Bachelor | 0.067103266 | 0.025536077 | 1632 | 2.627782878 | 0.205 |
| Female, HS Diploma/GED - Female, <HS Diploma | 0.037013208 | 0.034439434 | 1632 | 1.074733336 | 0.987 |
| Female, Bachelor - Female, Graduate Degree | 0.015095885 | 0.015048218 | 1632 | 1.003167636 | 0.992 |
| Female, <HS Diploma - Female, Some College | -0.035027848 | 0.031107686 | 1632 | -1.126019094 | 0.982 |
| Female, <HS Diploma - Female, Graduate Degree | 0.045185944 | 0.0339604 | 1632 | 1.330548052 | 0.947 |
| Female, <HS Diploma - Female, Bachelor | 0.030090059 | 0.033397386 | 1632 | 0.900970472 | 0.996 |
| **Household Income Post-hoc Contrasts** | **Estimate** | **SE** | **df** | **T** | **p value** |
| Male, [≥50K & <100K] - Male, [≥100K] | 0.025915204 | 0.014247822 | 1632 | 1.818888854 | 0.454 |
| Male, [≥50K & <100K] - Male, [<50K] | -0.026240225 | 0.016487585 | 1632 | -1.591514192 | 0.604 |
| Male, [<50K] - Male, [≥100K] | 0.052155429 | 0.018074982 | 1632 | 2.88550384 | **0.046** |
| Female, [≥50K & <100K] - Female, [≥100K] | 0.072689736 | 0.014854516 | 1632 | 4.893443719 | **<0.001** |
| Female, [≥50K & <100K] - Female, [<50K] | -0.033340875 | 0.017338917 | 1632 | -1.922892615 | 0.388 |
| Female, [<50K] - Female, [≥100K] | 0.106030611 | 0.018983784 | 1632 | 5.58532543 | **<0.001** |

**Supplementary Table 5.**

Post-hoc comparisons for sociodemographic differences in DHEA levels for males and females.

| **Weight Status Post-hoc Contrasts** | **Estimate** | **SE** | **df** | **T** | **p value** |
| --- | --- | --- | --- | --- | --- |
| Male, Underweight - Male, Overweight | -16.58239623 | 5.693141064 | 444 | -2.912697234 | 0.072 |
| Male, Underweight - Male, Obese | -30.74821024 | 5.71370703 | 444 | -5.381481773 | **<0.001** |
| Male, Overweight - Male, Obese | -14.16581401 | 3.41467523 | 444 | -4.148509904 | **0.001** |
| Male, Healthy Weight - Male, Underweight | 6.863542346 | 5.275370492 | 444 | 1.301054088 | 0.898 |
| Male, Healthy Weight - Male, Overweight | -9.718853884 | 2.754573493 | 444 | -3.528260875 | **0.011** |
| Male, Healthy Weight - Male, Obese | -23.88466789 | 2.757044332 | 444 | -8.663142488 | **<0.001** |
| Female, Underweight - Female, Overweight | -18.78851391 | 6.06851541 | 444 | -3.096064299 | **0.043** |
| Female, Underweight - Female, Obese | -28.55809052 | 6.107054839 | 444 | -4.676245961 | **<0.001** |
| Female, Overweight - Female, Obese | -9.769576617 | 3.70606357 | 444 | -2.636106055 | 0.146 |
| Female, Healthy Weight - Female, Underweight | 8.062109672 | 5.577817999 | 444 | 1.445387726 | 0.835 |
| Female, Healthy Weight - Female, Overweight | -10.72640423 | 2.964179499 | 444 | -3.61867567 | **0.008** |
| Female, Healthy Weight - Female, Obese | -20.49598085 | 2.975623141 | 444 | -6.887962582 | **<0.001** |
| **Race/Ethnicity Post-hoc Contrasts** | **Estimate** | **SE** | **df** | **T** | **p value** |
| Male, White - Male, Other | 0.828784706 | 3.548720293 | 444 | 0.233544669 | 1.000 |
| Male, White - Male, Black | -18.766333 | 3.739827297 | 444 | -5.017967812 | **<0.001** |
| Male, White - Male, Asian | -8.313563016 | 6.517689114 | 444 | -1.275538442 | 0.959 |
| Male, Hispanic - Male, White | 4.624960987 | 3.075137487 | 444 | 1.503985108 | 0.890 |
| Male, Hispanic - Male, Other | 5.453745693 | 4.116893722 | 444 | 1.324723459 | 0.948 |
| Male, Hispanic - Male, Black | -14.14137201 | 4.030658334 | 444 | -3.508452178 | **0.018** |
| Male, Hispanic - Male, Asian | -3.688602029 | 6.919000101 | 444 | -0.533112007 | 1.000 |
| Male, Black - Male, Other | 19.5951177 | 4.593222829 | 444 | 4.266093424 | **0.001** |
| Male, Black - Male, Asian | 10.45276998 | 7.296312401 | 444 | 1.432609983 | 0.916 |
| Male, Asian - Male, Other | 9.142347722 | 7.142408628 | 444 | 1.280009055 | 0.958 |
| Female, White - Female, Other | -2.234323322 | 3.648286526 | 444 | -0.612430878 | 1.000 |
| Female, White - Female, Black | -31.3229053 | 3.769044129 | 444 | -8.310570063 | **<0.001** |
| Female, White - Female, Asian | -13.1746971 | 6.570931929 | 444 | -2.00499674 | 0.596 |
| Female, Hispanic - Female, White | 9.485116697 | 3.142960229 | 444 | 3.017892689 | 0.079 |
| Female, Hispanic - Female, Other | 7.250793375 | 4.230493855 | 444 | 1.713935447 | 0.787 |
| Female, Hispanic - Female, Black | -21.83778861 | 4.081115746 | 444 | -5.350935863 | **<0.001** |
| Female, Hispanic - Female, Asian | -3.6895804 | 6.95574541 | 444 | -0.530436378 | 1.000 |
| Female, Black - Female, Other | 29.08858198 | 4.703098107 | 444 | 6.18498303 | **<0.001** |
| Female, Black - Female, Asian | 18.14820821 | 7.318836129 | 444 | 2.479657679 | 0.282 |
| Female, Asian - Female, Other | 10.94037377 | 7.2116775 | 444 | 1.517035915 | 0.885 |
| **Highest Education Post-hoc Contrasts** | **Estimate** | **SE** | **df** | **T** | **p value** |
| Male, Some College - Male, Bachelor | -3.013237611 | 2.96735483 | 444 | -1.015462519 | 0.991 |
| Male, HS Diploma/GED - Male, Some College | 7.010304885 | 4.056273833 | 444 | 1.728262236 | 0.779 |
| Male, HS Diploma/GED - Male, Graduate Degree | 1.744669323 | 4.516108615 | 3905 | 0.386321382 | 1.000 |
| Male, HS Diploma/GED - Male, Bachelor | 3.997067274 | 4.436952328 | 444 | 0.900858738 | 0.996 |
| Male, HS Diploma/GED - Male, < HS Diploma | -2.097877206 | 6.461400373 | 3905 | -0.324678411 | 1.000 |
| Male, Bachelor - Male, Graduate Degree | -2.252397951 | 2.623258237 | 444 | -0.858626085 | 0.998 |
| Male, <HS Diploma - Male, Some College | 9.108182091 | 6.054023722 | 444 | 1.504484044 | 0.890 |
| Male, <HS Diploma - Male, Graduate Degree | 3.842546529 | 6.434321951 | 3905 | 0.597195254 | 1.000 |
| Male, <HS Diploma - Male, Bachelor | 6.09494448 | 6.36918686 | 444 | 0.956942325 | 0.994 |
| Female, Some College - Female, Graduate Degree | 2.912687659 | 3.278574014 | 444 | 0.888400764 | 0.997 |
| Female, Some College - Female, Bachelor | 0.947183925 | 3.131165512 | 444 | 0.30250203 | 1.000 |
| Female, HS Diploma/GED - Female, Some College | 9.385786772 | 4.502494458 | 444 | 2.084574864 | 0.539 |
| Female, HS Diploma/GED - Female, Graduate Degree | 12.29847443 | 4.978123042 | 444 | 2.47050431 | 0.287 |
| Female, HS Diploma/GED - Female, Bachelor | 10.3329707 | 4.840152636 | 444 | 2.134843976 | 0.504 |
| Female, HS Diploma/GED - Female, <HS Diploma | 11.92968592 | 6.44392504 | 444 | 1.851307371 | 0.702 |
| Female, Bachelor - Female, Graduate Degree | 1.965503735 | 2.708893353 | 444 | 0.725574424 | 0.999 |
| Female, <HS Diploma - Female, Some College | -2.54389915 | 5.660222185 | 444 | -0.449434504 | 1.000 |
| Female, <HS Diploma - Female, Graduate Degree | 0.368788509 | 6.174147284 | 444 | 0.059731084 | 1.000 |
| Female, <HS Diploma - Female, Bachelor | -1.596715226 | 6.033015178 | 444 | -0.264662889 | 1.000 |
| **Household Income Post-hoc Contrasts** | **Estimate** | **SE** | **df** | **T** | **p value** |
| Male, [≥50K & <100K] - Male, [≥100K] | 0.157694089 | 2.60149543 | 444 | 0.060616708 | 1.000 |
| Male, [≥50K & <100K] - Male, [<50K] | 1.064782723 | 3.044359431 | 444 | 0.349755917 | 0.999 |
| Male, [<50K] - Male, [≥100K] | -0.907088634 | 3.27478987 | 444 | -0.276991401 | 1.000 |
| Female, [≥50K & <100K] - Female, [≥100K] | -5.503657567 | 2.669410028 | 444 | -2.06175054 | 0.309 |
| Female, [≥50K & <100K] - Female, [<50K] | -3.029711267 | 3.113355369 | 444 | -0.973133776 | 0.926 |
| Female, [<50K] - Female, [≥100K] | -2.4739463 | 3.386179033 | 444 | -0.730601151 | 0.978 |

**Supplementary Table 6.**

Post-hoc comparisons for sociodemographic differences in Testosterone levels for males and females.

| **Weight Status Post-hoc Contrasts** | **Estimate** | **SE** | **df** | **T** | **p value** |
| --- | --- | --- | --- | --- | --- |
| Male, Underweight - Male, Overweight | -5.95064516 | 2.064988587 | 444 | -2.881684285 | 0.079 |
| Male, Underweight - Male, Obese | -8.524506208 | 2.071882573 | 444 | -4.114377097 | **0.001** |
| Male, Overweight - Male, Obese | -2.573861048 | 1.228464962 | 444 | -2.095184746 | 0.420 |
| Male, Healthy Weight - Male, Underweight | 2.962357874 | 1.913230084 | 444 | 1.54835422 | 0.781 |
| Male, Healthy Weight - Male, Overweight | -2.988287286 | 0.990502961 | 444 | -3.016939274 | 0.054 |
| Male, Healthy Weight - Male, Obese | -5.562148334 | 0.997932116 | 444 | -5.573674048 | **<0.001** |
| Female, Underweight - Female, Overweight | -4.385115287 | 2.170640392 | 444 | -2.020194273 | 0.469 |
| Female, Underweight - Female, Obese | -6.278965499 | 2.186498203 | 444 | -2.871699364 | 0.081 |
| Female, Overweight - Female, Obese | -1.893850212 | 1.347993749 | 444 | -1.404939907 | 0.855 |
| Female, Healthy Weight - Female, Underweight | 1.836873758 | 1.991419979 | 444 | 0.922393959 | 0.984 |
| Female, Healthy Weight - Female, Overweight | -2.548241529 | 1.077460478 | 444 | -2.365044084 | 0.261 |
| Female, Healthy Weight - Female, Obese | -4.442091741 | 1.081818261 | 444 | -4.106134924 | **0.001** |
| **Race/Ethnicity Post-hoc Contrasts** | **Estimate** | **SE** | **df** | **T** | **p value** |
| Male, White - Male, Other | -0.567476716 | 1.293470013 | 444 | -0.438724292 | 1.000 |
| Male, White - Male, Black | -5.968717022 | 1.377201115 | 444 | -4.333947278 | **0.001** |
| Male, White - Male, Asian | -0.442864298 | 2.358944135 | 444 | -0.187738358 | 1.000 |
| Male, Hispanic - Male, White | 0.0852526 | 1.125756902 | 444 | 0.075729138 | 1.000 |
| Male, Hispanic - Male, Other | -0.482224116 | 1.506832668 | 444 | -0.320024994 | 1.000 |
| Male, Hispanic - Male, Black | -5.883464422 | 1.491991363 | 444 | -3.943363593 | **0.004** |
| Male, Hispanic - Male, Asian | -0.357611698 | 2.507729125 | 444 | -0.142603798 | 1.000 |
| Male, Black - Male, Other | 5.401240306 | 1.690367292 | 444 | 3.195305738 | **0.048** |
| Male, Black - Male, Asian | 5.525852724 | 2.652872318 | 444 | 2.082969726 | 0.541 |
| Male, Asian - Male, Other | -0.124612418 | 2.5868349 | 444 | -0.048171771 | 1.000 |
| Female, White - Female, Other | -0.98226556 | 1.333883984 | 444 | -0.736395048 | 0.999 |
| Female, White - Female, Black | -11.15122096 | 1.391996151 | 444 | -8.010956745 | **<0.001** |
| Female, White - Female, Asian | -7.649634597 | 2.368552489 | 444 | -3.229666488 | **0.043** |
| Female, Hispanic - Female, White | 1.205609825 | 1.157581735 | 444 | 1.041490021 | 0.990 |
| Female, Hispanic - Female, Other | 0.223344265 | 1.548663172 | 444 | 0.144217457 | 1.000 |
| Female, Hispanic - Female, Black | -9.945611133 | 1.503458937 | 444 | -6.615153157 | **<0.001** |
| Female, Hispanic - Female, Asian | -6.444024773 | 2.508850597 | 444 | -2.568516746 | 0.236 |
| Female, Black - Female, Other | 10.1689554 | 1.725980309 | 444 | 5.891698384 | **<0.001** |
| Female, Black - Female, Asian | 3.50158636 | 2.650871406 | 444 | 1.320918983 | 0.949 |
| Female, Asian - Female, Other | 6.667369037 | 2.600358924 | 444 | 2.56401875 | 0.238 |
| **Highest Education Post-hoc Contrasts** | **Estimate** | **SE** | **df** | **T** | **p value** |
| Male, Some College - Male, Graduate Degree | -0.864161597 | 1.095798647 | 444 | -0.788613491 | 0.999 |
| Male, Some College - Male, Bachelor | -0.558473289 | 1.086787626 | 444 | -0.513875274 | 1.000 |
| Male, HS Diploma/GED - Male, Some College | 2.8666115 | 1.471375797 | 444 | 1.94825245 | 0.636 |
| Male, HS Diploma/GED - Male, Graduate Degree | 2.002449903 | 1.633796225 | 3798 | 1.225642386 | 0.968 |
| Male, HS Diploma/GED - Male, Bachelor | 2.308138211 | 1.6110482 | 444 | 1.432693454 | 0.916 |
| Male, HS Diploma/GED - Male, <HS Diploma | -2.739069572 | 2.334327391 | 3798 | -1.173387068 | 0.976 |
| Male, Bachelor - Male, Graduate Degree | -0.305688308 | 0.953562268 | 444 | -0.320575088 | 1.000 |
| Male, <HS Diploma - Male, Some College | 5.605681072 | 2.188593078 | 444 | 2.56131719 | 0.239 |
| Male, <HS Diploma - Male, Graduate Degree | 4.741519475 | 2.326680766 | 3798 | 2.037890003 | 0.572 |
| Male, <HS Diploma - Male, Bachelor | 5.047207783 | 2.307824394 | 444 | 2.186998194 | 0.467 |
| Female, Some College - Female, Graduate Degree | 1.462781427 | 1.208516427 | 444 | 1.210394326 | 0.971 |
| Female, Some College - Female, Bachelor | 0.29310249 | 1.154687705 | 444 | 0.253837024 | 1.000 |
| Female, HS Diploma/GED - Female, Some College | 1.838429945 | 1.640764132 | 444 | 1.120471803 | 0.982 |
| Female, HS Diploma/GED - Female, Graduate Degree | 3.301211372 | 1.823978308 | 444 | 1.80989618 | 0.729 |
| Female, HS Diploma/GED - Female, Bachelor | 2.131532436 | 1.772815306 | 444 | 1.202343204 | 0.972 |
| Female, HS Diploma/GED - Female, <HS Diploma | 1.404992854 | 2.386295392 | 444 | 0.588775748 | 1.000 |
| Female, Bachelor - Female, Graduate Degree | 1.169678937 | 0.995000848 | 444 | 1.175555718 | 0.976 |
| Female, <HS Diploma - Female, Some College | 0.433437091 | 2.115783614 | 444 | 0.204858894 | 1.000 |
| Female, <HS Diploma - Female, Graduate Degree | 1.896218518 | 2.309772293 | 444 | 0.820954742 | 0.998 |
| Female, <HS Diploma - Female, Bachelor | 0.726539582 | 2.257983788 | 444 | 0.321764747 | 1.000 |
| **Household Income Posthoc Contrasts** | **Estimate** | **SE** | **df** | **T** | **p value** |
| Male, [≥50K & <100K] - Male, [≥100K] | 0.494077059 | 0.94677543 | 444 | 0.521852431 | 0.995 |
| Male, [≥50K & <100K] - Male, [<50K] | -0.411396591 | 1.107488704 | 444 | -0.37146798 | 0.999 |
| Male, [<50K] - Male, [≥100K] | 0.905473651 | 1.194352342 | 444 | 0.758129422 | 0.974 |
| Female, [≥50K & <100K] - Female, [≥100K] | -0.74195744 | 0.979369852 | 444 | -0.757586563 | 0.974 |
| Female, [≥50K & <100K] - Female, [<50K] | 0.004678149 | 1.146227899 | 444 | 0.004081343 | 1.000 |
| Female, [<50K] - Female, [≥100K] | -0.746635589 | 1.248912012 | 444 | -0.597828816 | 0.991 |

**Supplementary Table 7.**

Post-hoc comparisons for sociodemographic differences in Estradiol levels for females only.

| **Weight Status Post-hoc Contrasts** | **Estimate** | **SE** | **df** | **T** | **p value** |
| --- | --- | --- | --- | --- | --- |
| Healthy Weight - Underweight | -0.01179736 | 0.064517394 | 145 | -0.18285549 | 0.998 |
| Healthy Weight - Overweight | -0.034451723 | 0.034166244 | 145 | -1.00835559 | 0.745 |
| Healthy Weight - Obese | -0.06694689 | 0.034611356 | 145 | -1.934246367 | 0.218 |
| Underweight - Overweight | -0.022654363 | 0.070065307 | 145 | -0.323332105 | 0.988 |
| Underweight - Obese | -0.05514953 | 0.070749778 | 145 | -0.779501109 | 0.864 |
| Overweight - Obese | -0.032495167 | 0.042888065 | 145 | -0.757673882 | 0.873 |
| **Race/Ethnicity Post-hoc Contrasts** | **Estimate** | **SE** | **df** | **T** | **p value** |
| Hispanic - White | 0.051923952 | 0.038738373 | 145 | 1.340375117 | 0.667 |
| Hispanic - Black | -0.114579605 | 0.048893122 | 1699 | -2.343470838 | 0.132 |
| Hispanic - Asian | 0.047013807 | 0.081520429 | 1699 | 0.576711964 | 0.978 |
| Hispanic - Other | -0.034765779 | 0.050042789 | 145 | -0.694721057 | 0.957 |
| White - Black | -0.166503557 | 0.043821445 | 145 | -3.799590735 | **0.002** |
| White - Asian | -0.004910145 | 0.077203146 | 145 | -0.063600322 | 1.000 |
| White - Other | -0.086689731 | 0.042122815 | 145 | -2.058023188 | 0.244 |
| Black - Asian | 0.161593412 | 0.085636591 | 1699 | 1.886966895 | 0.325 |
| Black - Other | 0.079813826 | 0.054235923 | 145 | 1.471604443 | 0.583 |
| Asian - Other | -0.081779586 | 0.08374465 | 145 | -0.976535045 | 0.865 |
| **Highest Education Post-hoc Contrasts** | **Estimate** | **SE** | **df** | **T** | **p value** |
| HS Diploma/GED - <HS Diploma | -0.010871147 | 0.074380474 | 1699 | -0.146155919 | 1.000 |
| HS Diploma/GED - Some College | -0.036616897 | 0.052151869 | 1699 | -0.702120518 | 0.956 |
| HS Diploma/GED - Bachelor | -0.108435389 | 0.056238634 | 1699 | -1.928129842 | 0.303 |
| HS Diploma/GED - Graduate Degree | -0.118352217 | 0.057922861 | 1699 | -2.043272998 | 0.246 |
| <HS Diploma - Some College | -0.025745751 | 0.065335287 | 1699 | -0.394055829 | 0.995 |
| <HS Diploma - Bachelor | -0.097564243 | 0.069983296 | 1699 | -1.394107567 | 0.632 |
| <HS Diploma - Graduate Degree | -0.10748107 | 0.07172816 | 1699 | -1.498450124 | 0.564 |
| Some College - Bachelor | -0.071818492 | 0.036499981 | 1699 | -1.967630942 | 0.282 |
| Some College - Graduate Degree | -0.08173532 | 0.038420759 | 1699 | -2.12737387 | 0.209 |
| Bachelor - Graduate Degree | -0.009916827 | 0.031456805 | 1699 | -0.315252219 | 0.998 |
| **Household Income Post-hoc Contrasts** | **Estimate** | **SE** | **df** | **T** | **p value** |
| [≥50K & <100K] - [<50K] | -0.070311266 | 0.036627929 | 1699 | -1.919608022 | 0.133 |
| [≥50K & <100K] - [≥100K] | -0.02380264 | 0.031121652 | 145 | -0.764825711 | 0.725 |
| [<50K] - [≥100K] | 0.046508626 | 0.039818834 | 145 | 1.168005701 | 0.474 |

**Supplementary Table 8.**

Latent factor 1 median loadings and 95% CI for males.

**Supplementary Table 9.**

Latent factor 2 median loadings and 95% CI for males.

**Supplementary Table 10.**

Latent factor 1 median loadings and 95% CI for females.

**Supplementary Table 11.**

Latent factor 2 median loadings and 95% CI for females.

**Supplementary Figure 1.**

DHEA and age associations in females (F) and males (M).


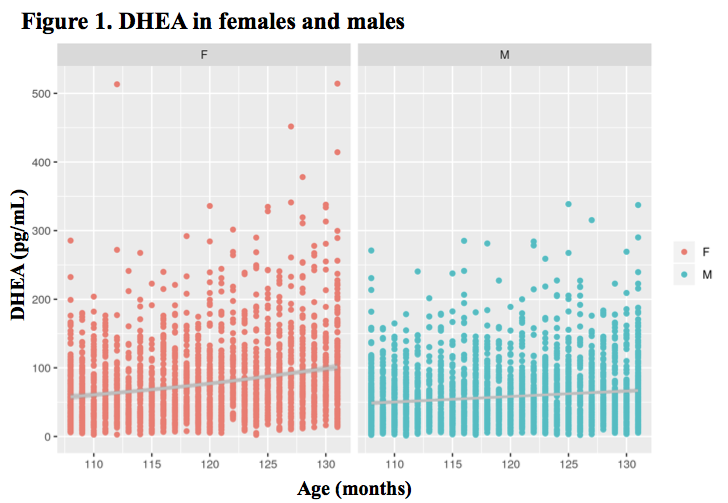


**Supplementary Figure 2.**

Testosterone and age associations in females (F) and males (M).


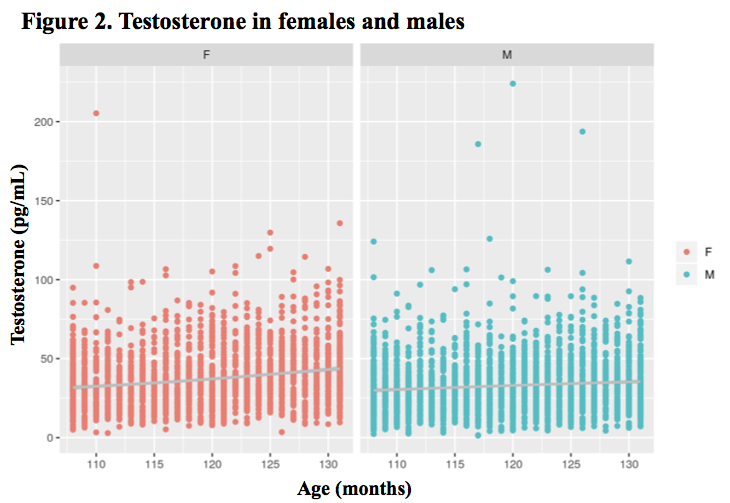


**Supplementary Figure 3.**

Estradiol and age associations in females (F) only.


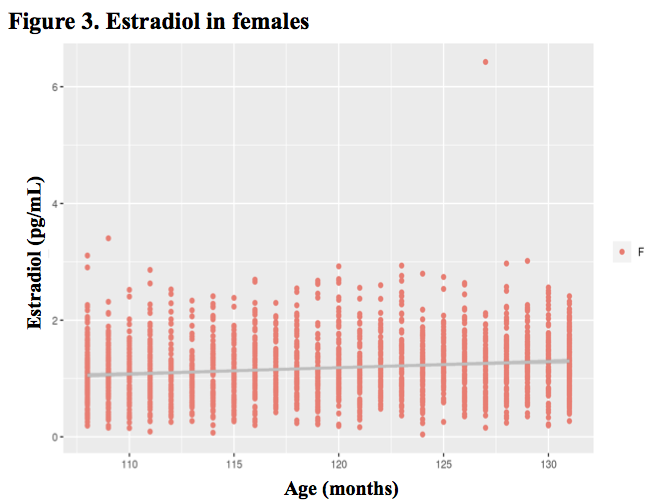

Supplement: Supplementary file 1 [file DataSheet_1.docx]
